# Supplementary material for: Temporal-spatial trends in childbirth in Ontario, Canada
Source: PLoS One. 2026 Mar 30;21(3):e0342215. doi: 10.1371/journal.pone.0342215 (PMC13035117; doi:10.1371/journal.pone.0342215)

# Supporting information

**Table A: Administrative codes.** Definitions of events based on International Classification of Diseases 10^th^ edition (ICD-10) diagnostic codes; Canadian Classification of Health Interventions (CCI) procedural codes; and select attributes (e.g., diagnostic code type, intervention status attributes). The Discharge Abstract Database (DAD) contains up to 25 diagnostic codes and up to 20 procedural codes.

**Table B: Description of procedure codes.** Description of the Canadian Classification of Interventions (CCI), used in this study.

**Table C: Description of diagnostic codes.** Description of the International Classification of Diseases, 10^th^ edition, used in this study.

**Table D: Definitions of indicators.**

**Table E: Trends of clinical characteristics over time.**

**Table F: Healthcare utilization patterns over time.**

**Table G: Outcomes over time.**

**Figure A: External validity checks compared with Statistics Canada data.** To guide the cohort creation process, we compared the numbers of live births, multiple gestation births, and stillbirths with Statistics Canada public reports.

# Table A: Administrative Codes

| Concept | Definitions [2,3] |
| --- | --- |
| Delivery abstract (person who delivered) | - - - Any diagnostic code of O10-O16, O21-O46, O48, O60-O75, O85-O92, O95, O98, O99 with a 6^th^ digit of 1 or 2 coded in any position; **or**     - Z37 coded in any position |
| Non-complex (low risk) delivery | - - - [Low-Risk Caesarean Sections \| CIHI](https://www.cihi.ca/en/indicators/low-risk-caesarean-sections) [4] |
| Complex delivery | - - - Adapted from [Low-Risk Caesarean Sections \| CIHI](https://www.cihi.ca/en/indicators/low-risk-caesarean-sections) [4]     - Any evidence of multiple gestation, gestational age <37 or >41 weeks, breech presentation, transverse/oblique lie, multiparity or unknown parity, or presence of any pre-existing delivery or fetal risks |
| Epidural | - - - Anesthetic technique code = 3; **or**     - 5LD20HAP1 in any position, not abandoned, not done OOH |
| Stillbirth, cadaver for donation, born *en route* | - - - Admission category = N (born *en route*), R (cadaveric donor), S (stillbirth) |
| Abortive procedure | - - - 5CA20, 5CA24, 5CA88, 5CA89, 5CA93 in any position, not abandoned, not done OOH; **or**     - O04 in any position |
| Caesarean section delivery | - - - 5MD60 in any position, not abandoned, not done OOH |
|  |  |
| Instrument-assisted delivery |  |
| Assisted delivery (forceps only) | - - - 5MD53KL, 5MD53KK, 5MD53KN, 5MD53KM, 5MD53KJ, 5MD53KH, 5MD53KS, 5MD53KP in any position, not abandoned, not done OOH; **or** |
| Assisted delivery (vacuum only) | - - - 5MD54 in any position, not abandoned, not done OOH |
| Assisted delivery (vacuum and forceps) | - - - 5MD55 in any position, not abandoned, not done OOH |
|  |  |
| Obstetrical surgical repair | - - - 5PC80JH, 5PC80JJ, 5PC80JR, 5PC80JQ, 5PC80JU, 5PC80JL, not abandoned and not done OOH |
| Instrument-assisted delivery | - - - 5MD53, 5MD54, 5M55, 5MD56NN, 5MD56PC, 5MD56NR, 5MD56PF, 5MD56NW, 5MD56PJ in any position and not abandoned or done OOH |
| Obstetrical hemorrhage | - - - O72.002, O72.102, O72.202, O90.202 in any position of diagnosis type M, 1, 2, W, X, Y |
| Obstetrical trauma | - - - O70.201, O70.211, O70.221, O72.31, O72.81, O72.91, O73.01, O71.181, O71.301, O71.401, O71.501, O71.601 in any position of diagnosis type M, 1, 2, W, X, Y |
| Blood transfusion | - - - Blood transfusion indicator |
| Birth trauma (N) | - - - P10-P15 in any position of diagnosis type M, 1, 2, W, X, Y |
| Brain damage (N) | - - - P10, P11.1, P11.2 in any position of diagnosis type M or 1; **and**     - Preterm and low birth weight (P07) I any position as diagnosis type M, 1, or 2 |
| Congenital malformations of the central nervous system | - - - Q00-Q07 in any position as diagnosis type M or 1 |
| Congenital malformations and deformations of the musculoskeletal system | - - - Q65-Q79 in any position as diagnosis type M or 1 |
|  |  |
| Previous, repeat, or undefined C-section | - - - Previous: O34.201, O66.401, O75.701 in any position; **or**     - Repeat: 5MD60 in any position coded as a repeat (status attribute N4, N6, RA, RB, or RC); **or**     - Undefined: [ 5MD60 in any position coded as undefined (status attribute Z); **and not**  O34.201, O66.401, O75.701 in any position ] |
| Termination of pregnancy | - - - P964 in any position |
| Out-of-hospital birth | - - - Z381, Z382, Z384, Z385, Z387, Z388 in any position |
| In-hospital birth | - - - Z380, Z383, Z386 in any position |
| NICU | - - - Special care unit number 1-6 includes 50, 51, 52, 53, or 98 |
| Midwife involved in delivery | - - - Intervention provider service (any position) = 11004 |
| Midwife involved in care | - - - Provider service (any position) = 11004 |
| ^a^ the 6^th^ digit of 1 means “delivered” and 2 means “delivered with complications”.  O10 – O16: Oedema, proteinuria and hypertensive disorders in pregnancy, childbirth and the puerperium  O20-O29: Other maternal disorders predominantly related to pregnancy  O30-O48: Maternal care related to the fetus and amniotic cavity and possible delivery problems  O60-O75: Complications of labour and delivery  O85-O92: Complications predominantly related to the puerperium  O94-O99: Other obstetric conditions, not elsewhere classified  Z73: Outcome of delivery  OOH – out-of-hospital | |

# Table B: Canadian Classification of Intervention (CCI) Procedure Codes

| **CCI Procedure Code** | **Long description** |
| --- | --- |
| 5CA20FK | Pharmacotherapy (in preparation for) termination of pregnancy using intracardiac/intrathoracic injection [e.g. KCL] |
| 5CA24CKBD | Preparation by dilating cervix (for) termination of pregnancy per orifice approach and balloon catheter |
| 5CA24CKW6 | Preparation by dilating cervix (for) termination of pregnancy per orifice approach and laminaria |
| 5CA88ALD2 | Pharmacological termination of pregnancy combined types of approaches salt solution |
| 5CA88ALG2 | Pharmacological termination of pregnancy combined types of approaches emollient |
| 5CA88ALI2 | Pharmacological termination of pregnancy combined types of approaches uterotonic agent |
| 5CA88ALM2 | Pharmacological termination of pregnancy combined types of approaches antimetabolite |
| 5CA88ALZ9 | Pharmacological termination of pregnancy combined types of approaches other abortifacient NEC |
| 5CA88CAI2 | Pharmacological termination of pregnancy oral approach uterotonic agent |
| 5CA88CAI5 | Pharmacological termination of pregnancy oral approach sex hormone and regulator |
| 5CA88CAM2 | Pharmacological termination of pregnancy oral approach antimetabolite |
| 5CA88CAZ9 | Pharmacological termination of pregnancy oral approach other abortifacient NEC |
| 5CA88CKI2 | Pharmacological termination of pregnancy per orifice [vaginal] approach uterotonic agent |
| 5CA88CKZ9 | Pharmacological termination of pregnancy per orifice [vaginal] approach other abortifacient NEC |
| 5CA88DAD2 | Pharmacological termination of pregnancy endoscopic (assisted) approach salt solution |
| 5CA88DAG2 | Pharmacological termination of pregnancy endoscopic (assisted) approach emollient |
| 5CA88DAM2 | Pharmacological termination of pregnancy endoscopic [assisted] approach antimetabolite |
| 5CA88DAZ9 | Pharmacological termination of pregnancy endoscopic (assisted) approach other abortifacient NEC |
| 5CA88HAD2 | Pharmacological termination of pregnancy percutaneous approach (e.g. intravenous injection into intraamniotic or extraamniotic sac) salt solution |
| 5CA88HAG2 | Pharmacological termination of pregnancy percutaneous approach (e.g. intravenous injection into intraamniotic or extraamniotic sac) emollient |
| 5CA88HAI2 | Pharmacological termination of pregnancy percutaneous approach (e.g. intravenous injection into intraamniotic or extraamniotic sac) uterotonic agent |
| 5CA88HAM2 | Pharmacological termination of pregnancy percutaneous approach (e.g. intravenous injection into intraamniotic or extraamniotic sac) antimetabolite |
| 5CA88HAZ9 | Pharmacological termination of pregnancy percutaneous approach (e.g. intravenous injection into intraamniotic or extraamniotic sac) other abortifacient NEC |
| 5CA89CK | Surgical termination of pregnancy hysterectomy vaginal approach |
| 5CA89DA | Surgical termination of pregnancy hysterotomy endoscopic approach |
| 5CA89FB | Surgical termination of pregnancy menstrual extraction or regulation (minisuction curettage) (that done with syringe) (HGC+) |
| 5CA89GA | Surgical termination of pregnancy vaginal approach dilation and durettage [D&C] |
| 5CA89GB | Surgical termination of pregnancy endoscopic approach hysterectomy |
| 5CA89GC | Surgical termination of pregnancy vaginal approach aspiration and curettage |
| 5CA89GD | Surgical termination of pregnancy vaginal approach dilation and evacuation [D&E] |
| 5CA89WJ | Surgical termination of pregnancy open approach hysterectomy |
| 5CA89WK | Surgical termination of pregnancy open approach hysterotomy |
| 5CA93EC | Surgical removal of extrauterine pregnancy open approach salpingostomy |
| 5CA93ED | Surgical removal of extrauterine pregnancy endoscopic approach salpingostomy |
| 5CA93EDAG | Surgical removal of extrauterine pregnancy endoscopic approach laser salpingostomy |
| 5CA93EF | Surgical removal of extrauterine pregnancy endoscopic approach with manual technique (fimbrial expression [e.g. milking]) |
| 5CA93EJ | Surgical removal of extrauterine pregnancy open approach partial salpingectomy |
| 5CA93EK | Surgical removal of extrauterine pregnancy endoscopic approach partial salpingectomy |
| 5CA93EKAG | Surgical removal of extrauterine pregnancy endoscopic approach laser partial salpingectomy |
| 5CA93EM | Surgical removal of extrauterine pregnancy open approach of other site (e.g. intraperitoneal intraligamentous site NEC) |
| 5CA93EQ | Surgical removal of extrauterine pregnancy endoscopic approach of other site (e.g. intraperitoneal intraligamentous site NEC) |
| 5CA93GB | Surgical removal of extrauterine pregnancy endoscopic approach total salpingectomy |
| 5CA93TC | Surgical removal of extrauterine pregnancy endoscopic approach salpingotomy |
| 5CA93TCAG | Surgical removal of extrauterine pregnancy endoscopic approach using laser salpingotomy |
| 5CA93UW | Surgical removal of extrauterine pregnancy open approach with manual technique (fimbrial expression [e.g. milking]) |
| 5CA93WJ | Surgical removal of extrauterine pregnancy open approach total salpingectomy |
| 5CA93WK | Surgical removal of extrauterine pregnancy open approach salpingotomy |
| 5LD20HAP1 | Intrapartum pharmacotherapy during active labour percutaneous approach [e.g. intramuscular intravenous subcutaneous intradermal] using general anesthetic |
| 5MD53JD | Forceps traction and rotation without episiotomy forceps rotation only with manually assisted delivery (e.g. DeLee key-in-lock maneuver) |
| 5MD53JE | Forceps traction and rotation with episiotomy forceps rotation only with manually assisted delivery (e.g. DeLee key-in-lock Maughan maneuver) |
| 5MD53KH | Forceps traction and rotation without episiotomy outlet forceps |
| 5MD53KJ | Forceps traction and rotation with episiotomy outlet forceps |
| 5MD53KK | Forceps traction and rotation without episiotomy low forceps (e.g. Pajot maneuver) |
| 5MD53KL | Forceps traction and rotation with episiotomy low forceps (e.g. Pajot maneuver) |
| 5MD53KM | Forceps traction and rotation without episiotomy mid forceps |
| 5MD53KN | Forceps traction and rotation with episiotomy mid forceps |
| 5MD53KP | Forceps traction and rotation without episiotomy double application of forceps (e.g. Scanzoni maneuver) |
| 5MD53KS | Forceps traction and rotation with episiotomy double application of forceps (e.g. Scanzoni maneuver) |
| 5MD54KH | Vacuum traction without episiotomy outlet vacuum traction |
| 5MD54KJ | Vacuum traction with episiotomy outlet vacuum traction |
| 5MD54KK | Vacuum traction without episiotomy low vacuum traction |
| 5MD54KL | Vacuum traction with episiotomy low vacuum traction |
| 5MD54KM | Vacuum traction without episiotomy mid vacuum traction |
| 5MD54KN | Vacuum traction with episiotomy mid vacuum traction |
| 5MD54NE | Vacuum traction without episiotomy NOS |
| 5MD54NF | Vacuum traction with episiotomy NOS |
| 5MD55KH | Combination of vacuum and forceps delivery without episiotomy outlet vacuum/forceps |
| 5MD55KJ | Combination of vacuum and forceps delivery with episiotomy outlet vacuum/forceps |
| 5MD55KK | Combination of vacuum and forceps delivery without episiotomy low vacuum/forceps |
| 5MD55KL | Combination of vacuum and forceps delivery with episiotomy low vacuum/forceps |
| 5MD55KM | Combination of vacuum and forceps delivery without episiotomy mid vacuum/forceps |
| 5MD55KN | Combination of vacuum and forceps delivery with episiotomy mid vacuum/forceps |
| 5MD55KQ | Combination of vacuum and forceps delivery without episiotomy vacuum with forceps NOS |
| 5MD55KR | Combination of vacuum and forceps delivery with episiotomy vacuum with forceps NOS |
| 5MD56NN | Breech delivery without episiotomy with forceps to aftercoming head [e.g. Piper] partial breech extraction [assisted breech delivery] |
| 5MD56NR | Breech delivery without episiotomy with forceps to aftercoming head [e.g. Piper] total breech extraction |
| 5MD56NW | Breech delivery without episiotomy with forceps to aftercoming head [e.g. Piper] unspecified breech extraction |
| 5MD56PC | Breech delivery with episiotomy with forceps to aftercoming head [e.g. Piper] partial breech extraction [assisted breech delivery] |
| 5MD56PF | Breech delivery with episiotomy with forceps to aftercoming head [e.g. Piper] total breech extraction |
| 5MD56PJ | Breech delivery with episiotomy with forceps to aftercoming head [e.g. Piper] unspecified breech extraction |
| 5MD60AA | Cesarean section without instrumentation lower segment transverse incision |
| 5MD60CB | Cesarean section with use of both vacuum and forceps cesarean hysterectomy |
| 5MD60CC | Cesarean section with use of both vacuum and forceps classical section [vertical incision in upper segment] |
| 5MD60CD | Cesarean section with use of both vacuum and forceps extraperitoneal section |
| 5MD60CE | Cesarean section with use of both vacuum and forceps inverted T incision |
| 5MD60CF | Cesarean section with use of both vacuum and forceps low segment transverse incision |
| 5MD60CG | Cesarean section with use of both vacuum and forceps other type of cesarean section NEC |
| 5MD60JW | Cesarean section with use of forceps lower segment transverse incision |
| 5MD60JX | Cesarean section with use of vacuum lower segment transverse incision |
| 5MD60JY | Cesarean section without instrumentation classical section [vertical incision in upper segment] |
| 5MD60JZ | Cesarean section with use of forceps classical section [vertical incision in upper segment] |
| 5MD60KA | Cesarean section with use of vacuum classical section [vertical incision in upper segment] |
| 5MD60KB | Cesarean section without instrumentation extraperitoneal section |
| 5MD60KC | Cesarean section with use of forceps extraperitoneal section |
| 5MD60KD | Cesarean section with use of vacuum extraperitoneal section |
| 5MD60KE | Cesarean hysterectomy without instrumentation |
| 5MD60KF | Cesarean laparotomy (for abdominal pregnancy) without instrumentation |
| 5MD60KG | Cesarean section without instrumentation inverted T incision |
| 5MD60KT | Cesarean section without instrumentation other type of Cesarean section NEC |
| 5MD60RA | Cesarean section with use of forceps inverted 'T' incision |
| 5MD60RB | Cesarean section with use of vacuum inverted T incision |
| 5MD60RC | Cesarean hysterectomy with use of forceps |
| 5MD60RD | Cesarean hysterectomy with use of vacuum |
| 5MD60RE | Cesarean laparotomy (for abdominal pregnancy) with use of forceps |
| 5MD60RF | Cesarean laparotomy (for abdominal pregnancy) with use of vacuum |
| 5MD60RG | Cesarean section with use of forceps other type of Cesarean section NEC |
| 5MD60RH | Cesarean section with use of vacuum other type of Cesarean section NEC |
| 5PC80JH | Surgical repair postpartum of obstetric laceration of corpus uteri [body of uterus] |
| 5PC80JJ | Surgical repair postpartum of current obstetric laceration of cervix occurring at vaginal delivery |
| 5PC80JL | Surgical repair postpartum of current obstetric laceration of broad ligament(s) of uterus |
| 5PC80JQ | Surgical repair postpartum of current obstetric laceration of rectum and sphincter ani |
| 5PC80JR | Surgical repair postpartum of current obstetric laceration of bladder and urethra |
| 5PC80JU | Surgical repair postpartum of current obstetric high vaginal laceration |

# Table C: International Classification of Diseases (ICD), 10^th^ edition

| **ICD10 Code** | **Long description** |
| --- | --- |
| O34201 | Maternal care for uterine scar due to previous caesarean section, delivered, with or without mention of antepartum condition |
| O66401 | Failed trial of labour following previous caesarean, unspecified, delivered, with or without mention of antepartum condition |
| O70211 | Third degree perineal laceration during delivery, type 3a, so described, delivered, with or without mention of antepartum condition |
| O70221 | Third degree perineal laceration during delivery, type 3b, so described, delivered, with or without mention of antepartum condition |
| O71181 | Other rupture of uterus during labour, delivered, with or without mention of antepartum condition |
| O71301 | Obstetric laceration of cervix, delivered, with or without mention of antepartum condition |
| O71401 | Obstetric high vaginal laceration, delivered, with or without mention of antepartum condition |
| O71501 | Other obstetric injury to pelvic organs, delivered, with or without mention of antepartum condition |
| O71601 | Obstetric damage to pelvic joints and ligaments, delivered, with or without mention of antepartum condition |
| O72002 | Third-stage haemorrhage, delivered, with mention o f postpartum complication |
| O72102 | Other immediate postpartum haemorrhage, delivered, with mention of postpartum complication |
| O72202 | Delayed and secondary postpartum haemorrhage, delivered, with mention of postpartum complication |
| O75701 | Vaginal delivery following previous caesarean sect ion, delivered, with or without mention of antepartum condition |
| O90202 | Haematoma of obstetric wound, delivered, with mention of postpartum complication |
| P070 | Extremely low birth weight |
| P071 | Other low birth weight |
| P072 | Extreme immaturity |
| P073 | Other preterm infants |
| P100 | Subdural haemorrhage due to birth injury |
| P101 | Cerebral haemorrhage due to birth injury |
| P102 | Intraventricular haemorrhage due to birth injury |
| P103 | Subarachnoid haemorrhage due to birth injury |
| P104 | Tentorial tear due to birth injury |
| P108 | Other intracranial lacerations and haemorrhages due to birth injury |
| P109 | Unspecified intracranial laceration and haemorrhage due to birth injury |
| P110 | Cerebral oedema due to birth injury |
| P111 | Other specified brain damage due to birth injury |
| P112 | Unspecified brain damage due to birth injury |
| P113 | Birth injury to facial nerve |
| P114 | Birth injury to other cranial nerves |
| P115 | Birth injury to spine and spinal cord |
| P119 | Birth injury to central nervous system, unspecified |
| P120 | Cephalhaematoma due to birth injury |
| P121 | Chignon due to birth injury |
| P122 | Epicranial subaponeurotic haemorrhage due to birth injury |
| P123 | Bruising of scalp due to birth injury |
| P124 | Monitoring injury of scalp of newborn |
| P128 | Other birth injuries to scalp |
| P129 | Birth injury to scalp, unspecified |
| P1300 | Linear skull fracture due to birth injury |
| P1301 | Depressed skull fracture due to birth injury |
| P1308 | Other/multiple skull fracture due to birth injury |
| P1309 | Unspecified skull fracture due to birth injury |
| P131 | Other birth injuries to skull |
| P132 | Birth injury to femur |
| P1330 | Birth injury to humerus |
| P1338 | Birth injury to other long bones |
| P134 | Fracture of clavicle due to birth injury |
| P138 | Birth injuries to other parts of skeleton |
| P139 | Birth injury to skeleton, unspecified |
| P140 | Erb's paralysis due to birth injury |
| P141 | Klumpke's paralysis due to birth injury |
| P142 | Phrenic nerve paralysis due to birth injury |
| P143 | Other brachial plexus birth injuries |
| P148 | Birth injuries to other parts of peripheral nervous system |
| P149 | Birth injury to peripheral nervous system, unspecified |
| P150 | Birth injury to liver |
| P151 | Birth injury to spleen |
| P152 | Sternomastoid injury due to birth injury |
| P153 | Birth injury to eye |
| P154 | Birth injury to face |
| P155 | Birth injury to external genitalia |
| P156 | Subcutaneous fat necrosis due to birth injury |
| P158 | Other specified birth injuries |
| P159 | Birth injury, unspecified |
| P964 | Termination of pregnancy, affecting fetus and newborn |
| Q000 | Anencephaly |
| Q001 | Craniorachischisis |
| Q002 | Iniencephaly |
| Q010 | Frontal encephalocele |
| Q011 | Nasofrontal encephalocele |
| Q012 | Occipital encephalocele |
| Q018 | Encephalocele of other sites |
| Q019 | Encephalocele, unspecified |
| Q02 | Microcephaly |
| Q030 | Malformations of aqueduct of Sylvius |
| Q031 | Atresia of foramina of Magendie and Luschka |
| Q038 | Other congenital hydrocephalus |
| Q039 | Congenital hydrocephalus, unspecified |
| Q040 | Congenital malformations of corpus callosum |
| Q041 | Arhinencephaly |
| Q042 | Holoprosencephaly |
| Q043 | Other reduction deformities of brain |
| Q044 | Septo-optic dysplasia |
| Q045 | Megalencephaly |
| Q046 | Congenital cerebral cysts |
| Q048 | Other specified congenital malformations of brain |
| Q049 | Congenital malformation of brain, unspecified |
| Q050 | Cervical spina bifida with hydrocephalus |
| Q051 | Thoracic spina bifida with hydrocephalus |
| Q052 | Lumbar spina bifida with hydrocephalus |
| Q053 | Sacral spina bifida with hydrocephalus |
| Q054 | Unspecified spina bifida with hydrocephalus |
| Q055 | Cervical spina bifida without hydrocephalus |
| Q056 | Thoracic spina bifida without hydrocephalus |
| Q057 | Lumbar spina bifida without hydrocephalus |
| Q058 | Sacral spina bifida without hydrocephalus |
| Q059 | Spina bifida, unspecified |
| Q060 | Amyelia |
| Q061 | Hypoplasia and dysplasia of spinal cord |
| Q062 | Diastematomyelia |
| Q063 | Other congenital cauda equina malformations |
| Q064 | Hydromyelia |
| Q068 | Other specified congenital malformations of spinal cord |
| Q069 | Congenital malformation of spinal cord, unspecified |
| Q070 | Arnold-Chiari syndrome |
| Q078 | Other specified congenital malformations of nervous system |
| Q079 | Congenital malformation of nervous system, unspecified |
| Q650 | Congenital dislocation of hip, unilateral |
| Q651 | Congenital dislocation of hip, bilateral |
| Q652 | Congenital dislocation of hip, unspecified |
| Q653 | Congenital subluxation of hip, unilateral |
| Q654 | Congenital subluxation of hip, bilateral |
| Q655 | Congenital subluxation of hip, unspecified |
| Q656 | Unstable hip |
| Q658 | Other congenital deformities of hip |
| Q659 | Congenital deformity of hip, unspecified |
| Q660 | Talipes equinovarus |
| Q661 | Talipes calcaneovarus |
| Q662 | Metatarsus varus |
| Q663 | Other congenital varus deformities of feet |
| Q664 | Talipes calcaneovalgus |
| Q665 | Congenital pes planus |
| Q666 | Other congenital valgus deformities of feet |
| Q667 | Pes cavus |
| Q668 | Other congenital deformities of feet |
| Q669 | Congenital deformity of feet, unspecified |
| Q670 | Facial asymmetry |
| Q671 | Compression facies |
| Q672 | Dolichocephaly |
| Q673 | Plagiocephaly |
| Q674 | Other congenital deformities of skull, face and ja w |
| Q675 | Congenital deformity of spine |
| Q676 | Pectus excavatum |
| Q677 | Pectus carinatum |
| Q678 | Other congenital deformities of chest |
| Q680 | Congenital deformity of sternocleidomastoid muscle |
| Q681 | Congenital deformity of hand |
| Q682 | Congenital deformity of knee |
| Q683 | Congenital bowing of femur |
| Q684 | Congenital bowing of tibia and fibula |
| Q685 | Congenital bowing of long bones of leg, unspecified |
| Q688 | Other specified congenital musculoskeletal deformities |
| Q690 | Accessory finger(s) |
| Q691 | Accessory thumb(s) |
| Q692 | Accessory toe(s) |
| Q699 | Polydactyly, unspecified |
| Q700 | Fused fingers |
| Q701 | Webbed fingers |
| Q702 | Fused toes |
| Q703 | Webbed toes |
| Q704 | Polysyndactyly |
| Q709 | Syndactyly, unspecified |
| Q710 | Congenital complete absence of upper limb(s) |
| Q711 | Congenital absence of upper arm and forearm with h and present |
| Q712 | Congenital absence of both forearm and hand |
| Q713 | Congenital absence of hand and finger(s) |
| Q714 | Longitudinal reduction defect of radius |
| Q715 | Longitudinal reduction defect of ulna |
| Q716 | Lobster-claw hand |
| Q718 | Other reduction defects of upper limb(s) |
| Q719 | Reduction defect of upper limb, unspecified |
| Q720 | Congenital complete absence of lower limb(s) |
| Q721 | Congenital absence of thigh and lower leg with foot present |
| Q722 | Congenital absence of both lower leg and foot |
| Q723 | Congenital absence of foot and toe(s) |
| Q724 | Longitudinal reduction defect of femur |
| Q725 | Longitudinal reduction defect of tibia |
| Q726 | Longitudinal reduction defect of fibula |
| Q727 | Split foot |
| Q728 | Other reduction defects of lower limb(s) |
| Q729 | Reduction defect of lower limb, unspecified |
| Q730 | Congenital absence of unspecified limb(s) |
| Q731 | Phocomelia, unspecified limb(s) |
| Q738 | Other reduction defects of unspecified limb(s) |
| Q740 | Other congenital malformations of upper limb(s), including shoulder girdle |
| Q741 | Congenital malformation of knee |
| Q742 | Other congenital malformations of lower limb(s), including pelvic girdle |
| Q743 | Arthrogryposis multiplex congenita |
| Q748 | Other specified congenital malformations of limb(s) |
| Q749 | Unspecified congenital malformation of limb(s) |
| Q750 | Craniosynostosis |
| Q751 | Craniofacial dysostosis |
| Q752 | Hypertelorism |
| Q753 | Macrocephaly |
| Q754 | Mandibulofacial dysostosis |
| Q755 | Oculomandibular dysostosis |
| Q758 | Other specified congenital malformations of skull and face bones |
| Q759 | Congenital malformation of skull and face bones, unspecified |
| Q760 | Spina bifida occulta |
| Q761 | Klippel-Feil syndrome |
| Q7620 | Congenital spondylolisthesis |
| Q7621 | Congenital spondylolysis |
| Q763 | Congenital scoliosis due to congenital bony malformation |
| Q764 | Other congenital malformations of spine, not associated with scoliosis |
| Q765 | Cervical rib |
| Q766 | Other congenital malformations of ribs |
| Q767 | Congenital malformation of sternum |
| Q768 | Other congenital malformations of bony thorax |
| Q769 | Congenital malformation of bony thorax, unspecified |
| Q770 | Achondrogenesis |
| Q771 | Thanatophoric short stature |
| Q772 | Short rib syndrome |
| Q773 | Chondrodysplasia punctata |
| Q774 | Achondroplasia |
| Q775 | Dystrophic dysplasia |
| Q776 | Chondroectodermal dysplasia |
| Q777 | Spondyloepiphyseal dysplasia |
| Q778 | Other osteochondrodysplasia with defects of growth of tubular bones and spine |
| Q779 | Osteochondrodysplasia with defects of growth of tubular bones and spine, unspecified |
| Q780 | Osteogenesis imperfecta |
| Q781 | Polyostotic fibrous dysplasia |
| Q782 | Osteopetrosis |
| Q783 | Progressive diaphyseal dysplasia |
| Q784 | Enchondromatosis |
| Q785 | Metaphyseal dysplasia |
| Q786 | Multiple congenital exostoses |
| Q788 | Other specified osteochondrodysplasias |
| Q789 | Osteochondrodysplasia, unspecified |
| Q790 | Congenital diaphragmatic hernia |
| Q791 | Other congenital malformations of diaphragm |
| Q792 | Exomphalos |
| Q793 | Gastroschisis |
| Q794 | Prune belly syndrome |
| Q795 | Other congenital malformations of abdominal wall |
| Q796 | Ehlers-Danlos syndrome |
| Q798 | Other congenital malformations of musculoskeletal system |
| Q799 | Congenital malformation of musculoskeletal system, unspecified |
| Z38000 | Singleton, born in hospital, product of both spontaneous (NOS) ovulation and conception, delivered vaginally |
| Z38001 | Singleton born in hospital, product of assisted re productive technology (ART), delivered vaginally |
| Z38010 | Singleton born in hospital, product of both spontaneous (NOS) ovulation and conception, delivered by caesarean |
| Z38011 | Singleton born in hospital, product of assisted re productive technology (ART), delivered by caesarean |
| Z38100 | Singleton, born outside hospital, product of both spontaneous (NOS) ovulation and conception |
| Z38101 | Singleton, born outside hospital, product of assisted reproductive technology (ART) |
| Z38200 | Singleton, unspecified as to place of birth, product of both spontaneous (NOS) ovulation and conception |
| Z38201 | Singleton, unspecified as to place of birth, product of assisted reproductive technology (ART) |
| Z38300 | Twin, born in hospital, product of both spontaneous (NOS) ovulation and conception, delivered vaginally |
| Z38301 | Twin, born in hospital, product of assisted reproductive technology (ART), delivered vaginally |
| Z38310 | Twin, born in hospital, product of both spontaneous (NOS) ovulation and conception, delivered by caesarean |
| Z38311 | Twin, born in hospital, product of assisted reproductive technology (ART), delivered by caesarean |
| Z38400 | Twin, born outside hospital, product of both spontaneous (NOS) ovulation and conception |
| Z38401 | Twin, born outside hospital, product of assisted reproductive technology (ART) |
| Z38500 | Twin, unspecified as to place of birth, product of both spontaneous (NOS) ovulation and conception |
| Z38501 | Twin, unspecified as to place of birth, product of assisted reproductive technology (ART) |
| Z38600 | Product of both spontaneous (NOS) ovulation and conception, triplet, born in hospital, delivered vaginally |
| Z38601 | Product of assisted reproductive technology (ART), triplet, born in hospital, delivered vaginally |
| Z38610 | Product of both spontaneous (NOS) ovulation and conception, triplet, born in hospital, delivered by caesarean |
| Z38611 | Product of assisted reproductive technology (ART), triplet, born in hospital, delivered by caesarean |
| Z38620 | Product of both spontaneous (NOS) ovulation and conception, quadruplet, born in hospital, delivered vaginally |
| Z38621 | Product of assisted reproductive technology (ART), quadruplet, born in hospital, delivered vaginally |
| Z38630 | Product of both spontaneous (NOS) ovulation and conception, quadruplet, born in hospital, delivered by caesarean |
| Z38631 | Product of assisted reproductive technology (ART), quadruplet, born in hospital, delivered by caesarean |
| Z38640 | Product of both spontaneous (NOS) ovulation and conception, quintuplet, born in hospital, delivered vaginally |
| Z38641 | Product of assisted reproductive technology (ART), quintuplet, born in hospital, delivered vaginally |
| Z38650 | Product of both spontaneous (NOS) ovulation and conception, quintuplet, born in hospital, delivered by caesarean |
| Z38651 | Product of assisted reproductive technology (ART), quintuplet, born in hospital, delivered by caesarean |
| Z38660 | Product of both spontaneous (NOS) ovulation and conception, sextuplet, born in hospital, delivered vaginally |
| Z38661 | Product of assisted reproductive technology (ART), sextuplet, born in hospital, delivered vaginally |
| Z38670 | Product of both spontaneous (NOS) ovulation and conception, sextuplet, born in hospital, delivered by caesarean |
| Z38671 | Product of assisted reproductive technology (ART), sextuplet, born in hospital, delivered by caesarean |
| Z38680 | Product of both spontaneous (NOS) ovulation and conception, other multiple birth, born in hospital, delivered vaginally |
| Z38681 | Product of assisted reproductive technology (ART), other multiple birth, born in hospital, delivered vaginally |
| Z38690 | Product of both spontaneous (NOS) ovulation and conception, other multiple birth, born in hospital, delivered by caesarean |
| Z38691 | Product of assisted reproductive technology (ART), other multiple birth, born in hospital, delivered by caesarean |
| Z38700 | Other multiple, born outside hospital, product of both spontaneous (NOS) ovulation and conception |
| Z38701 | Other multiple, born outside hospital, product of assisted reproductive technology (ART) |
| Z38800 | Other multiple, unspecified as to place of birth, product of both spontaneous (NOS) ovulation and conception |
| Z38801 | Other multiple, unspecified as to place of birth, product of assisted reproductive technology (ART) |

# Table D: Definitions of indicators

| Indicator | Denominator | Numerator |
| --- | --- | --- |
| **Delivery outcomes^a^** |  |  |
| Obstetric hemorrhage following non-instrumented vaginal delivery | Exclusions   - - - Instrument-assisted delivery     - Caesarean section delivery | Obstetric hemorrhage |
| Obstetric hemorrhage following instrument-assisted delivery or Caesarean delivery | Inclusions   - - - Instrument-assisted delivery (forceps only, vacuum only, or both); or     - Caesarean section delivery | Obstetric hemorrhage |
|  |  |  |
| Obstetric trauma during a non-instrumented vaginal delivery | Exclusions   - - - Instrument-assisted delivery     - Caesarean section delivery     - Abortive procedure | Inclusions   - - - Obstetric trauma; or     - Obstetric surgical repair |
| Obstetric trauma during an instrument-assisted vaginal delivery | Inclusions   - - - Instrument-assisted delivery (forceps only, vacuum only, or both)   Exclusions   - - - Caesarean section delivery     - Abortive procedure | Inclusions   - - - Obstetric trauma; or     - Obstetric surgical repair |
|  |  |  |
| Birth trauma during non-instrumented vaginal delivery | Exclusions (person who delivered)   - - - Instrument-assisted delivery     - Caesarean section delivery   Exclusions (newborn)   - - - Brain damage (N)     - Congenital malformations of the central nervous system     - Congenital malformations of the musculoskeletal system | Birth trauma |
| Birth trauma during instrument-assisted or Caesarean delivery | Inclusions (person who delivered)   - - - Instrument-assisted delivery     - Caesarean section delivery   Exclusions (newborn)   - - - Brain damage (N)     - Congenital malformations of the central nervous system     - Congenital malformations of the musculoskeletal system | Birth trauma |
|  |  |  |
| Epidural rate for all deliveries | Exclusions: none | Epidural |
| Epidural rate for vaginal deliveries | Exclusions   - - - Caesarean section delivery | Epidural |
|  |  |  |
| Assisted delivery rate (overall) among *vaginal* deliveries | Exclusions   - - - Caesarean section delivery | Assisted delivery forceps only, vacuum only, or vacuum and forceps |
| Assisted delivery rate (*vacuum* extraction) among *vaginal* deliveries | Exclusions   - - - Caesarean section delivery | Assisted delivery vacuum only |
| Assisted delivery rate (*forceps*) among *vaginal* deliveries | Exclusions   - - - Caesarean section delivery | Assisted delivery forceps only |
|  |  |  |
| Total Caesarean section rate | Exclusions: none  Stratifications:   - - - Complicated delivery and not a complicated delivery | Caesarean section |
| Primary Caesarean section rate | Exclusions:   - - - Previous, repeat, or undefined C-section   Stratifications:   - - - Age <35 versus >=35 | Caesarean section |
| Repeat Caesarean section rate | Inclusions:   - - - Previous, repeat, or undefined C-section | Caesarean section |
|  |  |  |
| Mortality (person who delivered) | Exclusions: none | Death date from RPDB within 42 days of delivery date |
| Mortality (newborn) | Exclusions: none | Death date from RPDB within 42 days of delivery date |
|  |  |  |
| **Birth outcomes** |  |  |
| Low birth weight rate (<2,500 grams) | Exclusions   - - - Missing or invalid birth weight (0000, 0001, ZZZZ) | Birth weight <2,500 grams |
| Low birth weight rate (500 to 2,500 grams) | Exclusions   - - - Missing or invalid birth weight (0000, 0001, ZZZZ)     - Birth weight <500 grams | Birth weight <2,500 grams |
|  |  |  |
| Preterm birth rate (<37 weeks gestation) | Exclusions   - - - Missing or invalid birth weight (0000, 0001, ZZZZ)     - Birth weight <500 grams     - Invalid gestational age (blank, 98, 99, ZZ)     - Gestational age <22 or >43 | Gestational age <37 weeks |
| Small-for-gestational age | Exclusions   - - - Sex not M or F     - Missing or invalid birth weight (0000, 0001, ZZZZ) |  |
|  |  |  |
| **Other outcomes** |  |  |
| NICU (N) | Exclusions: none | NICU during admission |
| Midwife involved with person who delivered | Exclusions: none | Midwife involved in delivery or care |
| Travel distance | Exclusions   - - - Missing postal code     - Latitude and longitude unavailable from PCCF+ | Straight-line travel distance >50 km |
| Emergency department visit within 42 days of delivery | Exclusions: none | Number with at least 1 ED visit |
| Emergency department visit within 42 days of birth | Exclusions: none | Number with at least 1 ED visit |

^a^ abstracts are restricted to delivery abstracts for persons who delivered having sex encoded as F and an age at delivery between 10 and 55 years

^b^ abstracts are restricted to those having admit category N, out-of-hospital birth indicator absent, in-hospital birth present, termination of pregnancy absent, and age +/- 2 days relative to the admission date

# **Table E:** Trends of clinical characteristics over time

|  | **2010** | **2011** | **2012** | **2013** | **2014** | **2015** | **2016** | **2017** | **2018** | **2019** | **2020** | **2021** | **2022** | **2023** | **p** |
| --- | --- | --- | --- | --- | --- | --- | --- | --- | --- | --- | --- | --- | --- | --- | --- |
| **N births** | **133,957** | **133,883** | **134,719** | **132,229** | **131,974** | **130,960** | **131,449** | **131,049** | **130,445** | **130,690** | **127,276** | **132,892** | **127,480** | **127,660** |  |
| Non-complex | 22.22% | 22.21% | 22.16% | 22.03% | 21.40% | 20.62% | 19.75% | 18.71% | 17.86% | 17.06% | 17.18% | 16.65% | 16.48% | 16.85% | *** |
|  |  |  |  |  |  |  |  |  |  |  |  |  |  |  |  |
| Complex | 70.39% | 70.42% | 70.76% | 70.64% | 71.41% | 72.02% | 72.99% | 73.93% | 74.40% | 74.71% | 74.17% | 74.66% | 74.99% | 73.84% | *** |
| Breech presentation | 4.89% | 4.78% | 4.80% | 4.83% | 4.74% | 4.68% | 4.73% | 5.02% | 4.92% | 4.95% | 4.93% | 4.99% | 5.12% | 5.27% | *** |
| Transverse/oblique lie | 0.50% | 0.47% | 0.48% | 0.46% | 0.42% | 0.48% | 0.49% | 0.49% | 0.51% | 0.58% | 0.52% | 0.55% | 0.46% | 0.58% | *** |
| Diabetes (pre-existing or gestational) | 5.56% | 5.62% | 6.08% | 6.07% | 6.69% | 7.34% | 8.11% | 9.22% | 9.48% | 10.40% | 10.39% | 10.24% | 10.53% | 11.08% | *** |
| Hypertension (pre-existing or gestational) | 4.75% | 4.74% | 4.20% | 4.09% | 4.01% | 4.25% | 4.17% | 4.31% | 4.50% | 4.67% | 5.23% | 5.12% | 5.20% | 5.40% | *** |
| Obesity | 1.62% | 1.95% | 2.20% | 2.29% | 2.41% | 2.89% | 3.47% | 3.83% | 4.14% | 4.31% | 4.45% | 4.37% | 4.25% | 4.62% | *** |
| Pre-eclampsia or eclampsia | 1.14% | 1.03% | 1.48% | 1.72% | 1.84% | 1.99% | 2.17% | 2.08% | 2.20% | 2.12% | 2.18% | 2.20% | 2.36% | 2.56% | *** |
| Venous complications | 0.03% | 0.03% | 0.03% | 0.04% | 0.04% | 0.04% | 0.04% | 0.04% | 0.03% | 0.05% | 0.05% | 0.05% | 0.04% | 0.05% | 0.0002 |
| Liver disorders | 0.43% | 0.59% | 0.67% | 0.79% | 0.97% | 1.06% | 1.21% | 1.23% | 1.22% | 1.50% | 1.43% | 1.31% | 1.35% | 1.16% | *** |
| Other specified pregnancy-related conditions | 0.46% | 0.45% | 0.43% | 0.43% | 0.44% | 0.41% | 0.45% | 0.53% | 0.47% | 0.59% | 0.59% | 0.61% | 0.54% | 0.61% | *** |
| Anesthesia-related complications during pregnancy | 0.01% | 0.01% | 0.01% | 0.01% | 0.01% | 0.01% | 0.01% | 0.01% | 0.01% | 0.01% | 0.01% | 0.01% | 0.01% | 0.01% | 0.13 |
| Pelvic organ abnormality | 12.94% | 12.93% | 12.75% | 12.77% | 12.83% | 12.72% | 12.75% | 13.36% | 13.64% | 13.80% | 14.09% | 14.27% | 14.55% | 14.54% | *** |
| Fetal abnormality and damage | 0.58% | 0.49% | 0.51% | 0.54% | 0.59% | 0.70% | 0.67% | 0.70% | 0.68% | 0.68% | 0.66% | 0.62% | 0.59% | 0.56% | *** |
| Other fetal problems | 4.35% | 4.56% | 4.59% | 4.96% | 5.14% | 5.61% | 6.25% | 6.75% | 7.46% | 8.04% | 8.64% | 9.42% | 9.37% | 9.86% | *** |
| Polyhydramnios | 0.58% | 0.57% | 0.64% | 0.66% | 0.64% | 0.76% | 0.86% | 0.87% | 0.93% | 1.00% | 1.04% | 1.04% | 0.98% | 0.92% | *** |
| Oligohydramnios and other amniotic fluid and membrane disorders | 1.62% | 1.66% | 1.70% | 1.84% | 1.99% | 1.91% | 1.87% | 1.81% | 1.76% | 1.54% | 1.51% | 1.47% | 1.46% | 1.38% | *** |
| Placental disorders | 0.55% | 0.60% | 0.56% | 0.55% | 0.56% | 0.64% | 0.69% | 0.82% | 0.90% | 0.84% | 0.79% | 0.98% | 0.83% | 0.87% | *** |
| Placenta previa | 0.65% | 0.67% | 0.74% | 0.70% | 0.71% | 0.70% | 0.73% | 0.74% | 0.78% | 0.81% | 0.78% | 0.75% | 0.73% | 0.82% | *** |
| Placental abruption | 0.96% | 0.95% | 0.95% | 0.99% | 0.96% | 1.02% | 1.02% | 1.04% | 1.01% | 1.01% | 0.96% | 1.00% | 1.06% | 1.00% | 0.007 |
| Antepartum hemorrhage | 0.22% | 0.23% | 0.26% | 0.29% | 0.32% | 0.29% | 0.28% | 0.31% | 0.30% | 0.33% | 0.29% | 0.33% | 0.31% | 0.29% | *** |
| Uterine rupture | 0.09% | 0.08% | 0.08% | 0.08% | 0.09% | 0.10% | 0.12% | 0.10% | 0.10% | 0.12% | 0.11% | 0.12% | 0.11% | 0.14% | *** |
| Obstetric embolism | 0.02% | 0.02% | 0.01% | 0.02% | 0.01% | 0.02% | 0.03% | 0.03% | 0.03% | 0.03% | 0.03% | 0.02% | 0.03% | 0.02% | 0.007 |
| Herpes | 0.09% | 0.11% | 0.09% | 0.08% | 0.10% | 0.10% | 0.11% | 0.12% | 0.12% | 0.10% | 0.23% | 0.51% | 1.80% | 0.48% | *** |
| HIV | 0.02% | 0.02% | 0.03% | 0.01% | 0.02% | 0.02% | 0.01% | 0.01% | 0.01% | 0.02% | 0.01% | 0.01% | 0.02% | 0.02% | 0.05 |
| Other disease | 4.90% | 5.65% | 5.86% | 6.19% | 6.27% | 7.25% | 7.91% | 8.50% | 9.13% | 9.30% | 9.74% | 9.91% | 9.89% | 10.72% | *** |
| HELLP | - | - | 0.37% | 0.52% | 0.51% | 0.51% | 0.51% | 0.47% | 0.52% | 0.48% | 0.46% | 0.45% | 0.47% | 0.50% | *** |
| *** p<0.0001  IQR – interquartile range | | | | | | | | | | | | | | | |

# **Table F:** Healthcare utilization patterns over time

|  | **2010** | **2011** | **2012** | **2013** | **2014** | **2015** | **2016** | **2017** | **2018** | **2019** | **2020** | **2021** | **2022** | **2023** | **p** |
| --- | --- | --- | --- | --- | --- | --- | --- | --- | --- | --- | --- | --- | --- | --- | --- |
| **N births** | **133,957** | **133,883** | **134,719** | **132,229** | **131,974** | **130,960** | **131,449** | **131,049** | **130,445** | **130,690** | **127,276** | **132,892** | **127,480** | **127,660** |  |
| Hospital LOS (hours) for PWD |  |  |  |  |  |  |  |  |  |  |  |  |  |  |  |
| Median (IQR) – all deliveries | 51.7 (36.8, 72.1) | 51.1 (36.7, 70.0) | 50.4 (36.3, 66.8) | 49.3 (35.8, 63.6) | 48.4 (35.5, 61.8) | 47.4 (35.1, 59.5) | 46.5 (34.8, 58.1) | 46.4 (34.8, 57.6) | 46.2 (34.8, 56.9) | 45.9 (34.8, 56.4) | 43.4 (33.7, 54.6) | 42.6 (33.5, 54.1) | 42.2 (33.4, 53.9) | 42.7 (33.7, 54.1) |  |
| Median (IQR) – vaginal | 43 (34, 56) | 42 (34, 55) | 42 (33, 54) | 41 (33, 53) | 40 (33, 52) | 40 (33, 51) | 39 (32, 50) | 39 (32, 50) | 39 (33, 50) | 39 (33, 49) | 38 (32, 49) | 38 (32, 48) | 38 (32, 48) | 38 (32, 48) |  |
| Median (IQR) – Cesarean delivery | 75 (57, 87) | 74 (56, 85) | 71 (55, 83) | 66 (54, 80) | 63 (53, 79) | 60 (53, 77) | 59 (52, 76) | 57 (52, 75) | 56 (52, 73) | 56 (51, 72) | 54 (47, 67) | 53 (44, 65) | 53 (42, 64) | 53 (43, 65) |  |
|  |  |  |  |  |  |  |  |  |  |  |  |  |  |  |  |
| Travel >50km | 4.57% | 4.51% | 4.48% | 4.59% | 4.62% | 4.76% | 4.84% | 5.04% | 5.20% | 5.17% | 5.38% | 5.67% | 5.98% | 5.90% | *** |
| Midwife involved in care | 7.44% | 8.01% | 8.27% | 9.28% | 9.98% | 10.60% | 10.98% | 11.51% | 12.22% | 12.36% | 12.15% | 11.99% | 12.29% | 12.30% | *** |
|  |  |  |  |  |  |  |  |  |  |  |  |  |  |  |  |
| Epidural rate (all) | 52.40% | 53.08% | 54.04% | 54.81% | 55.10% | 52.11% | 51.07% | 51.33% | 51.55% | 52.36% | 53.88% | 53.68% | 52.88% | 53.21% | *** |
| Epidural rate (vaginal) | 62.1% | 62.7% | 63.7% | 64.6% | 65.1% | 62.0% | 60.8% | 61.7% | 62.1% | 63.3% | 65.9% | 66.2% | 65.7% | 66.4% | *** |
|  |  |  |  |  |  |  |  |  |  |  |  |  |  |  |  |
| Vaginal delivery, with assistance (any) | 13.60% | 13.31% | 13.32% | 13.17% | 12.68% | 12.63% | 12.46% | 12.35% | 12.12% | 12.66% | 12.79% | 12.90% | 12.57% | 12.60% | *** |
| Forceps only | 3.20% | 3.24% | 3.11% | 3.11% | 2.89% | 2.96% | 2.91% | 2.85% | 2.79% | 2.84% | 2.95% | 2.99% | 2.94% | 2.84% | *** |
| Vacuum only | 9.82% | 9.54% | 9.69% | 9.46% | 9.22% | 9.06% | 9.06% | 9.05% | 8.88% | 9.31% | 9.36% | 9.45% | 9.24% | 9.36% | *** |
|  |  |  |  |  |  |  |  |  |  |  |  |  |  |  |  |
| Induced labor | 24.25% | 24.15% | 23.68% | 24.33% | 24.82% | 26.27% | 27.21% | 28.93% | 30.71% | 32.85% | 34.14% | 34.54% | 33.73% | 35.01% | *** |
|  |  |  |  |  |  |  |  |  |  |  |  |  |  |  |  |
| Cesarean section delivery | 29.3% | 29.5% | 29.2% | 28.7% | 28.7% | 28.9% | 29.1% | 29.7% | 30.3% | 30.8% | 32.1% | 32.9% | 33.9% | 34.6% | *** |
| Non-complex | 16.5% | 17.1% | 16.6% | 15.7% | 15.3% | 15.8% | 15.7% | 15.7% | 16.3% | 16.1% | 17.8% | 18.2% | 19.3% | 19.3% | *** |
| Complex | 33.3% | 33.3% | 33.0% | 32.7% | 32.6% | 32.6% | 32.7% | 33.2% | 33.7% | 34.2% | 35.6% | 36.3% | 37.1% | 38.3% | *** |
|  |  |  |  |  |  |  |  |  |  |  |  |  |  |  |  |
| ICU for PWD | 0.21% | 0.22% | 0.23% | 0.24% | 0.22% | 0.23% | 0.24% | 0.24% | 0.22% | 0.24% | 0.18% | 0.24% | 0.23% | 0.22% | 0.84 |
| NICU for newborn | 12.21% | 12.03% | 11.94% | 12.44% | 12.83% | 12.67% | 12.68% | 12.76% | 12.87% | 12.99% | 13.11% | 12.87% | 12.47% | 12.80% | *** |
| Newborn transfer | 2.57% | 2.52% | 2.67% | 2.65% | 2.73% | 2.70% | 2.79% | 2.88% | 2.77% | 2.57% | 2.67% | 2.77% | 2.79% | 2.76% | *** |
| *** p<0.0001  LOS –length of stay; IQR – interquartile range; PWD – person who delivered; ICU – intensive care unit; NICU – neonatal intensive care unit | | | | | | | | | | | | | | | |

# **Table G:** Outcomes over time

|  | **2010** | **2011** | **2012** | **2013** | **2014** | **2015** | **2016** | **2017** | **2018** | **2019** | **2020** | **2021** | **2022** | **2023** | **p** |
| --- | --- | --- | --- | --- | --- | --- | --- | --- | --- | --- | --- | --- | --- | --- | --- |
| **N births** | **133,957** | **133,883** | **134,719** | **132,229** | **131,974** | **130,960** | **131,449** | **131,049** | **130,445** | **130,690** | **127,276** | **132,892** | **127,480** | **127,660** |  |
| Obstetric hemorrhage | 0.48% | 0.50% | 0.54% | 0.51% | 0.50% | 0.56% | 0.48% | 0.50% | 0.53% | 0.55% | 0.50% | 0.54% | 0.51% | 0.55% | 0.05 |
| Vaginal, non-instrumented | 0.33% | 0.33% | 0.35% | 0.37% | 0.35% | 0.40% | 0.34% | 0.31% | 0.36% | 0.39% | 0.35% | 0.38% | 0.37% | 0.40% | 0.02 |
| Vaginal, instrumented | 0.69% | 0.78% | 0.82% | 0.74% | 0.76% | 0.80% | 0.71% | 0.80% | 0.79% | 0.79% | 0.71% | 0.76% | 0.71% | 0.74% | 0.53 |
|  |  |  |  |  |  |  |  |  |  |  |  |  |  |  |  |
| Obstetric trauma | 3.15% | 3.21% | 3.43% | 3.38% | 3.32% | 3.31% | 3.32% | 3.29% | 3.24% | 3.36% | 3.51% | 3.41% | 3.49% | 3.64% | *** |
| Vaginal, non-instrumented | 2.72% | 2.74% | 3.05% | 2.90% | 2.84% | 2.81% | 2.86% | 2.88% | 2.84% | 3.03% | 3.24% | 3.17% | 3.34% | 3.61% | *** |
| Instrumented or Cesarean | 13.10% | 13.25% | 13.73% | 14.14% | 14.27% | 14.35% | 14.31% | 14.24% | 14.61% | 14.42% | 14.86% | 14.25% | 14.99% | 14.73% | *** |
|  |  |  |  |  |  |  |  |  |  |  |  |  |  |  |  |
| Birth trauma | 0.64% | 0.59% | 0.58% | 0.66% | 0.73% | 0.73% | 0.83% | 0.83% | 0.85% | 0.82% | 0.93% | 0.92% | 0.89% | 0.96% | *** |
| Vaginal, non-instrumented | 0.40% | 0.42% | 0.37% | 0.40% | 0.46% | 0.48% | 0.49% | 0.50% | 0.56% | 0.52% | 0.56% | 0.55% | 0.57% | 0.53% | *** |
| Instrumented or Cesarean | 1.01% | 0.87% | 0.93% | 1.10% | 1.16% | 1.13% | 1.39% | 1.37% | 1.31% | 1.27% | 1.47% | 1.44% | 1.33% | 1.54% | *** |
|  |  |  |  |  |  |  |  |  |  |  |  |  |  |  |  |
| SGA | 9.74% | 9.58% | 9.54% | 9.64% | 9.77% | 9.82% | 9.92% | 9.98% | 9.92% | 9.98% | 10.07% | 9.73% | 10.41% | 10.95% | *** |
| GA 21-36 weeks | 8.01% | 8.01% | 8.10% | 7.99% | 7.87% | 7.92% | 8.09% | 8.03% | 8.27% | 8.17% | 8.05% | 8.09% | 8.29% | 8.46% | *** |
| Low birth weight rate (up to 2,500g; excluding <500g) | 6.66% | 6.59% | 6.46% | 6.52% | 6.52% | 6.63% | 6.72% | 6.85% | 6.92% | 7.01% | 6.86% | 6.94% | 7.36% | 7.63% | *** |
|  |  |  |  |  |  |  |  |  |  |  |  |  |  |  |  |
| Emergency department (ED) visit | |  |  |  |  |  |  |  |  |  |  |  |  |  |  |
| within 42d of discharge (PWD) | 11.7% | 12.3% | 12.6% | 12.6% | 12.9% | 12.9% | 13.5% | 13.5% | 13.4% | 13.4% | 9.5% | 11.8% | 13.2% | 13.5% | *** |
| within 42d of discharge (newborn) | 9.0% | 9.1% | 9.1% | 8.8% | 8.8% | 8.8% | 8.7% | 8.8% | 8.6% | 8.9% | 7.6% | 8.4% | 8.2% | 8.7% | *** |
|  |  |  |  |  |  |  |  |  |  |  |  |  |  |  |  |
| Mortality |  |  |  |  |  |  |  |  |  |  |  |  |  |  |  |
| After delivery |  |  |  |  |  |  |  |  |  |  |  |  |  |  |  |
| 6 months, per 10K | 1.27 | 0.75 | 0.96 | 0.83 | 0.99 | 1.53 | 1.22 | 1.60 | 1.92 | 1.15 | 1.81 | 1.58 | 1.65 | 1.18 | 0.01 |
| 1 year, per 10K | 2.24 | 1.34 | 1.93 | 2.04 | 2.05 | 2.44 | 2.59 | 3.20 | 3.30 | 2.22 | 3.14 | 2.71 | 3.14 | NR | 0.0004 |
|  |  |  |  |  |  |  |  |  |  |  |  |  |  |  |  |
| After birth |  |  |  |  |  |  |  |  |  |  |  |  |  |  |  |
| 6 months, per 10K | 34.6 | 30.5 | 29.2 | 30.9 | 27.1 | 26.2 | 28.0 | 26.9 | 30.2 | 26.6 | 24.7 | 26.7 | 27.9 | 25.5 | *** |
| 1 year, per 10K | 36.8 | 33.0 | 31.5 | 32.4 | 28.8 | 27.9 | 30.1 | 29.0 | 32.4 | 29.0 | 25.6 | 29.2 | 30.1 | NR | *** |
| *** p<0.0001  IQR – interquartile range; SGA – small for gestational age; GA – gestational age; ED – emergency department; PWD – person who delivered; NR – not reportable (insufficient follow-up for 1-year mortality) | | | | | | | | | | | | | | | |

# Figure A: External validity checks compared with Statistics Canada

A) Number of live births


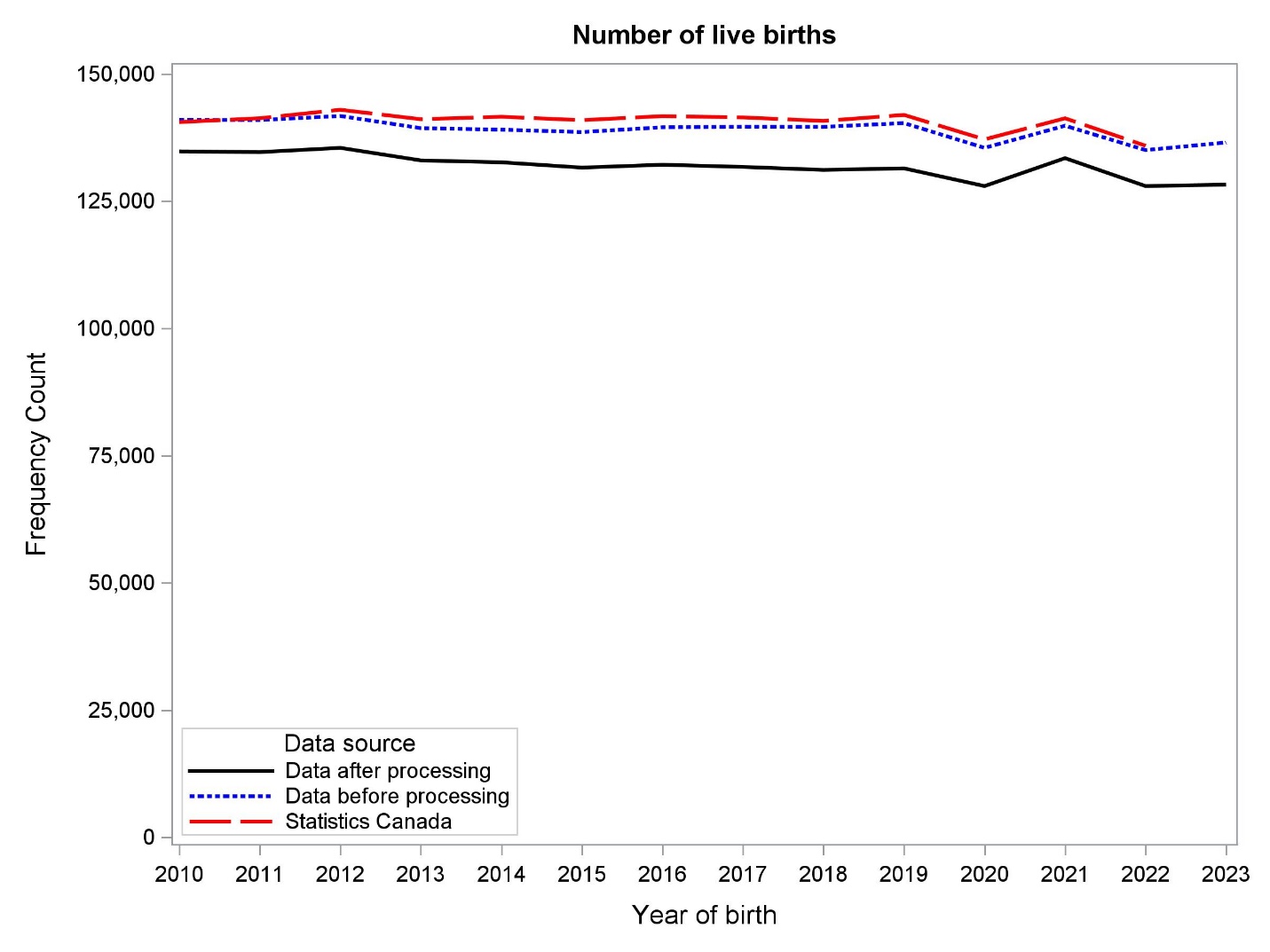


B) Number of multiple gestation births


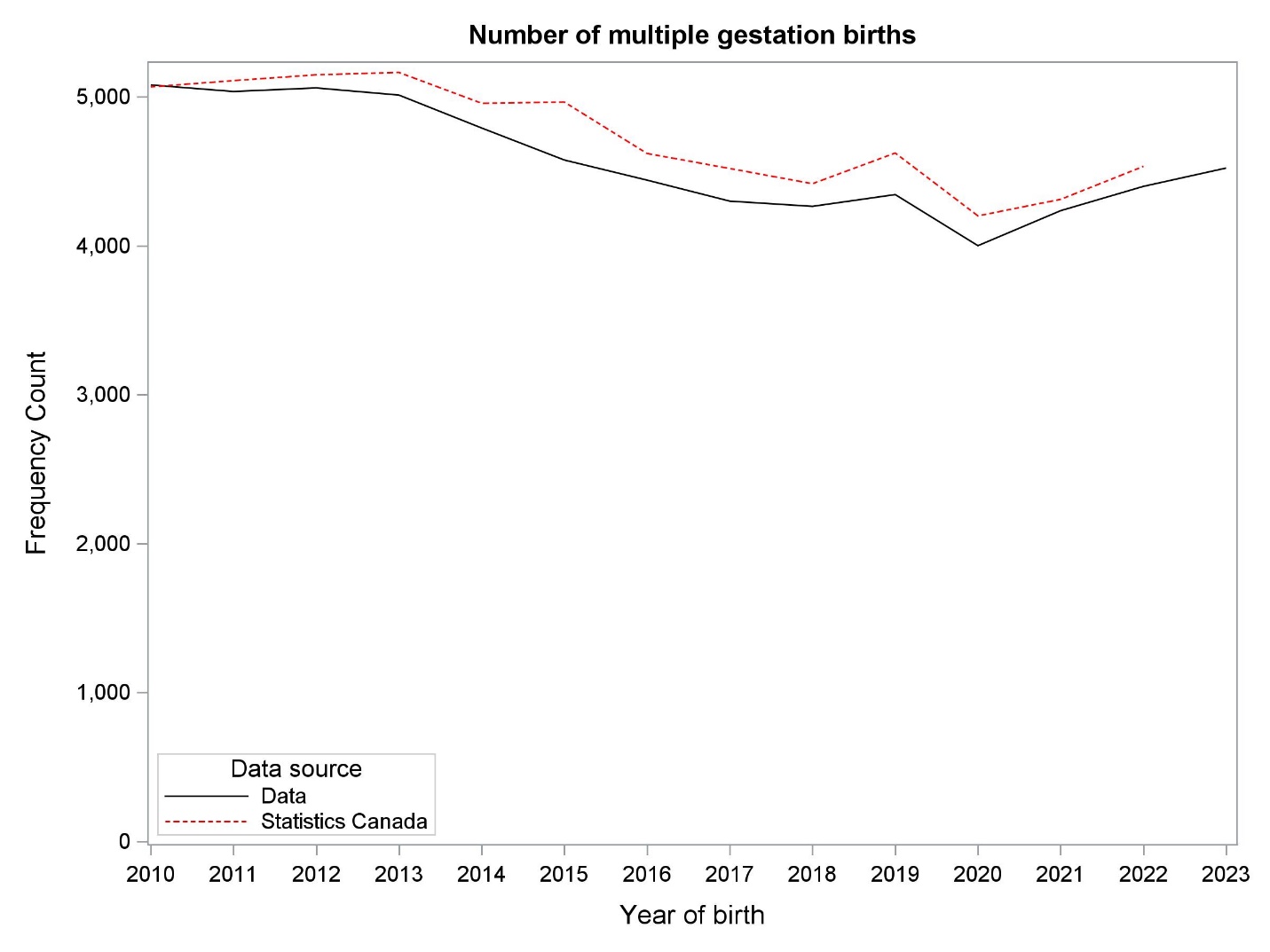


C) Number of still births


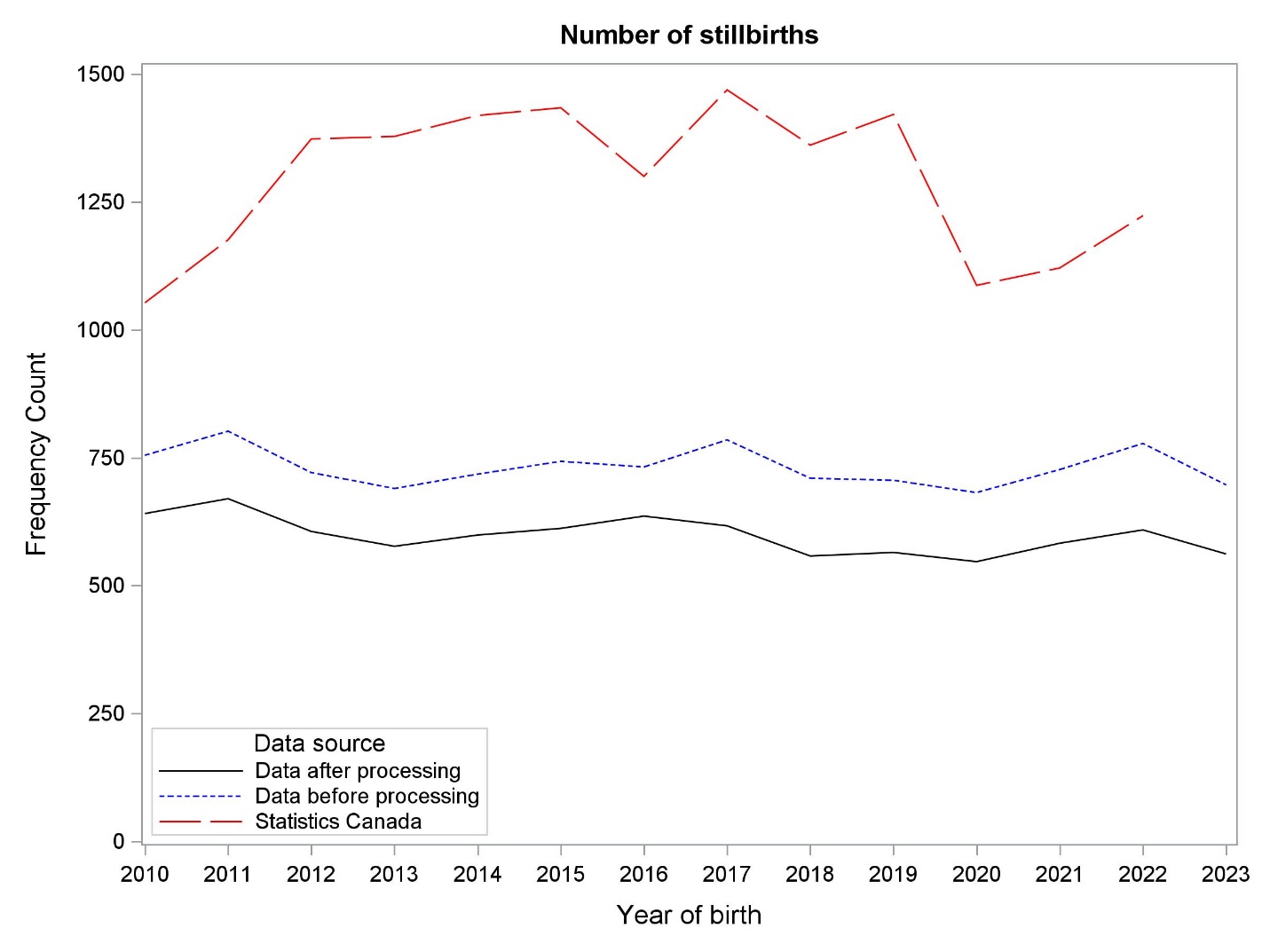

Supplement: S1 File — Administrative codes and definitions of indicators used in this study. Clinical characteristics, healthcare utilization patterns, and outcomes over time. (DOCX) [file pone.0342215.s001.docx]
