# Supplementary material for: Temporal-spatial trends in childbirth in Ontario, Canada
Source: PLoS One. 2026 Mar 30;21(3):e0342215. doi: 10.1371/journal.pone.0342215 (PMC13035117; doi:10.1371/journal.pone.0342215)
Supplement: S3 File — Additional details on data sources and linking newborn abstracts with persons who delivered. Data quality assessments are reported. (DOCX) [file pone.0342215.s003.docx]

Technical Appendix: Linking delivery and newborn abstracts using the hospital admission database in Ontario

# Introduction

The purpose of this Technical Appendix is to describe the process of linking abstracts of persons who delivered (PWD) to newborn abstracts to define cohorts for potential study. To be comprehensive of all deliveries/births in the province, we attempted to create three cohorts: 1) liveborn delivered; 2) liveborn not delivered; and 3) stillborn delivered. External validity checks involved comparing numbers with that reported by Statistics Canada.

# Methods

Inpatient hospital records were obtained from the Discharge Abstract Database (DAD) with admissions between 2010 and 2022.

## Data source for linkage

All hospitals in Ontario are mandated to report to the DAD for acute care, which is maintained by the Canadian Institute for Health Information (CIHI). CIHI also mandates that hospitals create an abstract with a unique chart number for all patients (including all liveborn babies as distinct from the PWD), recommending consistent formatting (e.g. on the use of leading zeros) and use of alphanumeric digits only (uppercase letters) of length 10 characters. For stillborns, the chart number is either the same as the PWD but with a prefix (‘Z’ for the first stillborn, ‘Y’, for the second, etc) or is a distinct chart number as is the case for newborns.

The maternal/newborn chart number (MNCN) is mandatory for all abstracts involving a live delivery (PWD and newborns) and follows the same rules as the chart number. One exception is that the MNCN on both the delivery and newborn abstract is blank for all stillborn, aborted, or undelivered cases. Second, the MNCN is blank for delivery and newborn abstracts when the newborn is delivered as a result of a failed medical abortion.

## Methodology for linking abstracts

Delivery abstracts and newborn abstracts were matched in three distinct steps

1. Liveborn deliveries born to PWDs following vaginal, cesarean, or water-based delivery
2. Stillborns following a vaginal, cesarean, or water-based delivery
3. Liveborns not delivered, following a failed medical abortion

Since liveborns born to persons following vaginal, cesarean, or water-based delivery are the most common, we started with this linkage with some learnings and data cleaning steps applied to other linkages.

Chart number: According to the CIHI methodology, chart numbers are mandatory data elements for all delivery and newborn abstracts (live or stillborn). Chart numbers can be alphanumeric strings and are unique by person and institution and should be consistent within the same institution. Leading zeros were removed before linking. Chart numbers were required to be not missing or invalid (e.g. not equal to ‘Y’, ‘/’, ‘9999’, ‘ZZZZZZZZZZ’, ‘,’).

# Results

## Cohort 1: Liveborns to PWD following vaginal, cesarean, or water delivery

Delivery abstracts were selected based on evidence of a live birth outcome (ICD-10 Z37 in any position) or an obstetrics code with a 1 or 2 coded in the 6^th^ position of the ICD-10 code to signify a liveborn delivery (Figure 1). Abstracts were excluded if there was evidence of a medical or surgical abortion and if the admission was categorized as N (newborn), S (stillborn), or R (cadaveric donor). Newborn abstracts had no evidence of a termination code (ICD-10 code P964 in any position) and had admission category N.

Delivery abstracts were removed if the age was not 10-55 years at the time of delivery (n=47) or not flagged with female sex (n=13). No newborn abstracts were removed due to age (**DQ1**).

| **DQ1:** **Age by source**  To determine age at delivery, we used the admission date and the date of birth either reported from the hospital Discharge Abstract Database (DAD) or the Registered Persons Database (RPDB). Excellent agreement was reported, but in all cases where the age of the PWD was >55 years from the RPDB, the age from DAD was appropriate for pregnancy. For all cases where the newborn was older than 0 days from RPDB, all ages from DAD were 0 days. We therefore used age as calculated using the date of birth from DAD. |
| --- |

After further restricting abstracts to those with a non-missing and valid chart number and MNCN (both delivery and newborn abstracts), there were 1,821,812 newborn abstracts left for matching. For all matches, the admission date for the baby was required to occur between the admission and discharge dates of the delivery abstract (inclusive), and all abstracts with an invalid or missing chart number were excluded.

### Match Step 1

The first match required both:

1. Chart number (delivery abstract) = MNCN (newborn abstract); and
2. MNCN (delivery abstract) = chart number (newborn abstract)

We did not require the institution number to match between the delivery and newborn abstracts (**DQ2**). A total 1,771,740 liveborn abstracts were matched to at least one delivery abstract.

| **DQ2:** **Institution number for matching**  After first matching delivery and newborn abstracts using the chart numbers and MNCN, matched pairs were dichotomized on whether or not the institution numbers were the same. Almost all matches had the same institution number (99.9%). After a review of the discrepancies, most discrepancies were due to the same institutions having different institution numbers (and names). These were associated with specific pairs where a change in hospital administration resulted in a change in administrative coding (e.g. “Rouge Valley Health System-Centenary” is the same location as “Scarborough Health Network-Centenary”). For cohorts that require matching on the same institution, the institution numbers were recoded to synergize such discrepancies (see Match 2: Stillborn and Match 3: Liveborn after Failed Abortion). |
| --- |

### Match Step 2

The second match required that:

1. Chart number (delivery abstract) = MNCN (newborn abstract);

A total 38,332 liveborn abstracts additional newborn abstracts were matched to at least one delivery abstract. It is expected that this step will capture the majority of the subsequent-borns when there are multiple births (e.g., the second newborn for a twin delivery). We compared the chart number of the newborn abstracts with the MNCN of the PWD (**DQ3**).

| **DQ3:** **Compare chart number of expected multiple gestation birth with MNCN of the PWD**  A data quality check comparing chart number (baby abstract) with MNCN (delivery abstract) indicated that most (74%) differed by a single character (e.g. an alphabetic prefix or the next chart number in sequence, as is expected for multiple births); 18% by two characters (e.g. an alphabetic prefix and the next chart number in sequence; the next number in sequence when it goes beyond a ten (e.g., XXXXXX59 -> XXXXXX60); or a possible typo due to a transposition); and 3% by three characters (no discernible pattern, but postal code for PWD and baby were identical for 94% of such instances). Notable hospital-specific variation was observed in chart-numbering practices. Thus, similarity between chart numbers was not used to verify the accuracy of matches, but provides general face validity for a multiple birth for the majority of instances. |
| --- |

### Match Step 3

The third match required that:

1. Chart number (newborn abstract) = MNCN (delivery abstract);

A total 4,840 liveborn abstracts additional newborn abstracts were matched to at least one delivery abstract. It is expected that this step will capture cases where there is some miscoding. Similar observations were observed as **DQ3** when comparing the similarity between the MNCN (baby) and the chart number (PWD), and notable hospital-specific coding patterns also emerged.

After removing records where the PWD or newborn had an invalid Ontario healthcard number (**DQ4**), de-duplication, and omitting matches where the delivery admit date was outside the study period, a total 1,717,343 liveborn PWD-baby matches were captured.

| **DQ4:** **Imputation of healthcard number**  To minimize the number of matches removed due to missing health card number, the healthcard number for the PWD and baby were imputed using all admission records matching the chart number and institution number (together these are unique identifiers for a specific person admitted to that hospital). While the same individual can have different chart numbers at different institutions, the same individually cannot have more than one chart number at the same institution as per CIHI-DADs manual. |
| --- |

As an additional data quality check, we examined the proportion of abstracts considered a multiple gestational birth for each step of the match (**DQ5**). As expected, the second match step captured most of the multiple gestation births.

| **DQ5:** **Multiple gestation deliveries by matching step**  A delivery was classified as a multiple gestation birth if either 1) the ICD-10 code Z383-Z388 was found in any position; 2) if any of the birth outcome codes Z372-Z379 were present in any position; or 3) a delivery occurred within 30 days of another delivery. From the final cohort (n=1,717,343), most of the abstracts captured in step 2 were twins or more:   \|  \| **Step 1** \| **Step 2** \| **Step 3** \| \| --- \| --- \| --- \| --- \| \| **Multiple gestation flag** \| 29,143 (1.74%) \| 29,526 (82.2%) \| 143 (3.12%) \| \| **Total** \| 1,676,838 \| 35,922 \| 4,583 \| |
| --- | --- | --- | --- | --- | --- | --- | --- | --- | --- | --- | --- | --- |

# Figure 1: Cohort creation (liveborn)

Extract all records from DAD between January 1, 2010 and December 31, 2023 (with 1 month buffer; pulled June 6, 2024

**(n=16,450,207 records)**

**Delivery abstract**

Inclusions: delivery/birth outcome

- - - Z37 in any position; or
    - O10-O16, O21-O46, O48, O60-O75, O85-O92, O95, O98, O99 with a 6^th^ digit of 1 or 2 coded in any position

Exclusions:

- - - Medical abortion (O04) or procedural abortion (5CA20, 5CA24, 5CA88, 5CA89, 5CA93) in any position
    - Admission categorized as newborn (N), stillborn (S), or deceased donor (R)
    - Missing or invalid discharge date

**Newborn abstract**

Inclusions:

- - - Admit category = N (newborn)
    - Not terminated (P964 in any position)

Exclusions:

- - - Missing or invalid discharge date

N=1,968,792 records

Exclude

- - - Invalid chart number (n<6)^a^
    - Age <10 (n=0) or >55 years (n=55)^b^
    - Non-female sex (n=15)
    - Exclude if invalid or missing MNCN (n=9,749)

^a^ Chart numbers are mandatory data elements for all persons who delivered (PWD) and babies (live or stillborn). Chart numbers can be alphanumeric strings and are unique by person and institution and should be consistent within the same institution. Leading zeros were removed before linking. Invalid chart numbers or MNCN were equal to ‘Y’, ‘/’, ‘9999’, ‘ZZZZZZZZZZ’, or ‘,’

^b^ age used from DAD rather than RPDB

^c^ data quality check on institution number identified 0.01% with a different institution number between mom and baby. Upon investigation, the majority of the discrepancies were the same institution, but following an administrative change (e.g. hospital merger). A similar trend was observed for step 2 (0.05% were mismatches) and step 3 (0.12% were mismatches).

^d^ a data quality check comparing chart number (baby abstract) with MNCN (mom abstract) indicated that most (74%) differed by a single character (e.g. an alphabetic prefix or the next chart number in sequence, as is expected for multiple births), 18% by two characters (e.g. an alphabetic prefix and/or the next chart number in sequence, a transposition), and 3% by three characters (no discernible pattern, but postal code for mom and baby were identical for 94% of instances). Similar observations were observed in step 3, but notable hospital-specific coding patterns emerged.

^e^ missing or invalid health insurance numbers were first imputed by linking to unique health insurance numbers from the admission database using institution number and chart number (unique patient identifier).

^f^ from footnote *c* above, institution numbers for institutions that changed names were re-coded and considered the same for the stillborn linkage, which requires the same institution number

^g^ a comparison of the chart numbers was performed using the Levenshtein edit distance between PWD and stillborn chart number. Most matches (68%) had an edit distance ≤3 and demonstrated alignment with the chart number for the matched PWD. However, there is sufficient evidence that chart number similarity not be used to adjudicate matches due to different hospital coding practices (e.g. the stillborn may be given their own chart instead of adopting the PWD’s chart and adding a prefix)

*should capture the multiple births (<https://www.cihi.ca/sites/default/files/document/dad-2016-2017-oydq-specs-final.pdf>)

N=1,929,241 records

Among unmatched liveborn abstracts

Exclude

- - - Invalid chart number (n<6)^a^
    - Age < 0 days (n<6) or >1 days (n=0)
    - Exclude if invalid or missing MNCN (n=11,020)^a^

Liveborn subset for matching

- - - N=1,957,767 newborn abstracts
    - N=1,919,420 delivery abstracts

Match PWD-to-liveborn baby (Step 1/3)

- - - Chart number (delivery) = MNCN (newborn);
    - MNCN (delivery) = chart number (newborn);
    - Baby abstract admission date during the delivery abstract admission and discharge dates, inclusive

Match PWD-to-liveborn baby (Step 2/3)*

- - - Chart number (delivery) = MNCN (newborn);
    - Baby abstract admission date during the delivery abstract admission and discharge dates, inclusive

Match PWD-to-liveborn baby (Step 3/3)

- - - MNCN (delivery) = chart number (newborn);
    - Baby abstract admission date during the delivery abstract admission and discharge dates, inclusive

Among unmatched liveborn abstracts

N=1,904,138 liveborn abstracts matched^c^

N=41,113 liveborn abstracts matched^c,d^

N=5,127 liveborn abstracts matched^c^

N=1,928,803 total liveborn abstracts matched

Exclude

- - - Delivery admit dates before January 1, 2010 or after December 31, 2023 (n=21,575)

Exclude records with a missing or invalid health insurance number^e^

N=1,846,607

Exclude

- - - Records for the same mother with a delivery >10 and <145 days after the previous delivery (n=12)
    - Remove n=918 duplicate records (same baby HIN matched to more than one PWD)
    - Remove n=260 duplicate records (different babies have the same HIN; likely miscoded twin records)

N=1,845,674 liveborn abstracts matched

Exclude

- - - Non-Ontario resident (n=8,564)
    - Out-of-hospital birth (n=448)

N=1,836,662

Final cohort

## Cohort 2: Stillborns to PWD following vaginal, cesarean, or water delivery

The cohort creation steps (Figure 2) began in the same fashion as the liveborn cohort (Figure 1) with the following exceptions:

- Delivery abstracts had no requirement for a valid or non-missing MNCN
- Stillborn abstracts had admission category S (stillborn)

After exclusions, a total 9,666 stillborn abstracts and 1,795,226 delivery abstracts were available for linkage. Since MNCN does not feature in the match, the delivery abstract was linked to the stillborn abstract using:

1. Same institution number (see **DQ2**)
2. Same postal code as per CIHI methodology

A total 8,699 stillborn abstracts were matched. Following de-duplication and removing abstracts with missing or invalid HIN [HINs only for PWDs wer imputed (see **DQ4**)], a total 7,817 matched pairs remained. Chart numbers were again not used to validate the accuracy of the matches (**DQ6**).

| **DQ6: Chart number coding for stillborns**  A comparison of the chart numbers was performed using the Levenshtein edit distance between delivery and stillborn chart number. Most matches (n=5,916 (68%) had an edit distance ≤3 and demonstrated alignment with the chart number for the matched PWD. However, there is sufficient evidence that chart number should not be used to adjudicate matches due to different hospital coding practices (e.g. the stillborn may be given their own chart instead of adopting the PWD’s chart and adding a prefix). |
| --- |

# Figure 2: Cohort creation (stillborn)

Extract all records from DAD between January 1, 2010 and December 31, 2023 (with 1 month buffer; pulled June 6, 2024

**(n=16,450,207 records)**

**Delivery abstract**

Inclusions: delivery/birth outcome

- - - Z37 in any position; or
    - O10-O16, O21-O46, O48, O60-O75, O85-O92, O95, O98, O99 with a 6^th^ digit of 1 or 2 coded in any position

Exclusions:

- - - Medical abortion (O04) or procedural abortion (5CA20, 5CA24, 5CA88, 5CA89, 5CA93) in any position
    - Admission categorized as newborn (N), stillborn (S), or deceased donor (R)
    - Missing or invalid discharge date
    - Invalid chart number (n<6)^a^

**Newborn abstract**

Inclusions:

- - - Admit category = S (stillborn)
    - Not terminated (P964 in any position)

Exclusions:

- - - Missing or invalid discharge date (n<6)
    - Invalid chart number (n<6)^a^

N=10,362 records

Match PWD-to-stillborn baby (Step 1/1)

- - - Same institution number^c^
    - Same postal code
    - Newborn abstract admission date during the delivery abstract admission and discharge dates, inclusive

Exclude

- - - Age <10 (n=0) or >55 years (n=55)^b^
    - Non-female sex (n=15)
    - Note: no exclusion based on MNCN

^a^ Chart numbers are mandatory data elements for all persons who delivered (PWD) and babies (live or stillborn). Chart numbers can be alphanumeric strings and are unique by person and institution and should be consistent within the same institution. Leading zeros were removed before linking. Chart numbers were required to be not missing or invalid (e.g. not equal to ‘Y’, ‘/’, ‘9999’, ‘ZZZZZZZZZZ’, ‘,’).

^b^ age used from DAD rather than RPDB

^c^ institution numbers for institutions that changed names were re-coded and considered the same for the stillborn linkage, which requires the same institution number

^d^ missing or invalid health insurance numbers were first imputed by linking to unique health insurance numbers from the admission database using institution number and chart number (unique patient identifier).

N=1,929,241 records

Exclude

- - - Records having MNCN (n=0)

Stillborn subset for matching

- - - N=10,362 stillborn abstracts
    - N=1,929,169 delivery abstracts

N=9,319 stillborn abstracts matched^g^

Exclude

- - - Delivery admit dates before January 1, 2010 or after December 31, 2022 (n=104)
    - 169, 19, 8, and 1 stillborns matched to two (338 records), three (57 records), four (32 records), and five (5 records) different PWD.

Exclude

- - - PWD having an invalid, missing, or multiple different health card numbers (PWD only)^e^

N=8,789

N=8,382 total stillborn abstracts

## Cohort 3: Liveborns following failed medial abortion

The cohort creation (Figure 3) was unique because PWD were not considered to have delivered the newborn (not a vaginal, cesarean, or water-birth as defined by CIHI).

Using CIHI methodology (<https://secure.cihi.ca/free_products/canadian-coding-standards-2022-en.pdf>), delivery abstracts were identified if medical abortion (ICD-10 code O04) was the most responsible diagnosis and a live birth outcome (ICD-10 code Z37) was present with diagnosis type 3 (“other” diagnosis type). The newborn abstract was required to have a termination (P964) as the most responsible diagnosis and a liveborn infant (Z38) of diagnosis type 0 (unhealthy infant).

A total 961 liveborn abstracts and 1,213 delivery abstracts were available for linkage. Linkage was conducted as per Match 2 (Stillborns), resulting in a total 710 liveborn abstracts matched to a delivery abstract.

# Figure 3: Cohort creation (liveborn after failed abortion)

Extract all records from DAD between January 1, 2010 and December 31, 2023 (with 1 month buffer; pulled June 6, 2024

**(n=16,450,207 records)**

**Delivery abstract**

Inclusions:

- - - Medical abortion (O04) as most responsible diagnosis
    - Birth outcome (Z37) of diagnosis type 3

**Newborn abstract**

Inclusions:

- - - Termination (P964) as most responsible diagnosis
    - Live birth (Z38) of diagnosis type 0

N=961 records

Match PWD-to-stillborn baby (Step 1/1)

- - - Same institution number^c^
    - Same postal code
    - Newborn abstract admission date during the delivery abstract admission and discharge dates, inclusive

Exclude

- - - Admit category N, S, or R (n=0)
    - Invalid discharge date (n=0)
    - Invalid chart number (n=0)^a^
    - Age <10 (n=0) or >55 years (n=0)^b^
    - Non-female sex (n=0)

^a^ Chart numbers are mandatory data elements for all persons who delivered (PWD) and newborns (live or stillborn). Chart numbers can be alphanumeric strings and are unique by person and institution and should be consistent within the same institution. Leading zeros were removed before linking. Chart numbers were required to be not missing or invalid (e.g. not equal to ‘Y’, ‘/’, ‘9999’, ‘ZZZZZZZZZZ’, ‘,’).

^b^ age used from DAD rather than RPDB

^c^ institution numbers for institutions that changed names were re-coded and considered the same for the linkage, which requires the same institution

^d^ missing or invalid health insurance numbers were first imputed by linking to unique health insurance numbers from the admission database using institution number and chart number (unique patient identifier).

N=1,213 records

Exclude

- - - Admit category not “S” (n=0)
    - Invalid discharge date (n=0)
    - Invalid chart number (n=0)^a^
    - Age > 0 days (n=0)

Stillborn subset for matching

- - - N=1,016 liveborn abstracts
    - N=1,276 delivery abstracts

N=790 abstracts matched^g^

Exclude

- - - Delivery admit dates before January 1, 2010 or after December 31, 2022 (n=6)
    - 0 liveborns matched to different PWD.(no duplicates)
    - PWD having an invalid, missing, or multiple different health card numbers (n=37)^d^

N=749 total liveborn abstracts after failed abortion

## Final Cohort

The final cohort consisted of 1,717,832 matched PWD-to-baby/stillborn abstracts after excluding records where the PWD’s postal code at the time of delivery (source: DAD) was not Ontario (did not start with K, L, M, N, or P), the province issuing the health card number was not Ontario, or the primary group responsible for payment of services was not 01 (the province).

N=7,817 stillborn deliveries

(Figure 2)

N=1,717,343 liveborn deliveries

(Figure 1)

N=710 born alive after failed abortion

(Figure 3)

Exclude records where

- Responsibility for payment not 01 or province issuing health card number is not Ontario (n=7,268)
- Non-Ontario mother postal code at the time of delivery (n=1,139)

N=1,725,870

Combine

N=1,717,832

(N = 1,709,388 liveborn delivery;

N = 7,736 stillborn delivery;

N = 708 liveborn after failed abortion)

## External validity checks

The number of total births was undercounted by a mean 8,516 births per year, which may be partly accounted for by the approximately 4,000 home births in Ontario per year (<https://www.ontariomidwives.ca/>), plus births performed in birthing centres (see main manuscript).

The number of multiple gestation births was similar between that reported by Statistics Canada and the admissions databases, although on average 160 fewer cases were captured (*Live Births and Fetal Deaths (Stillbirths), by Type of Birth (Single or Multiple)*, n.d.).


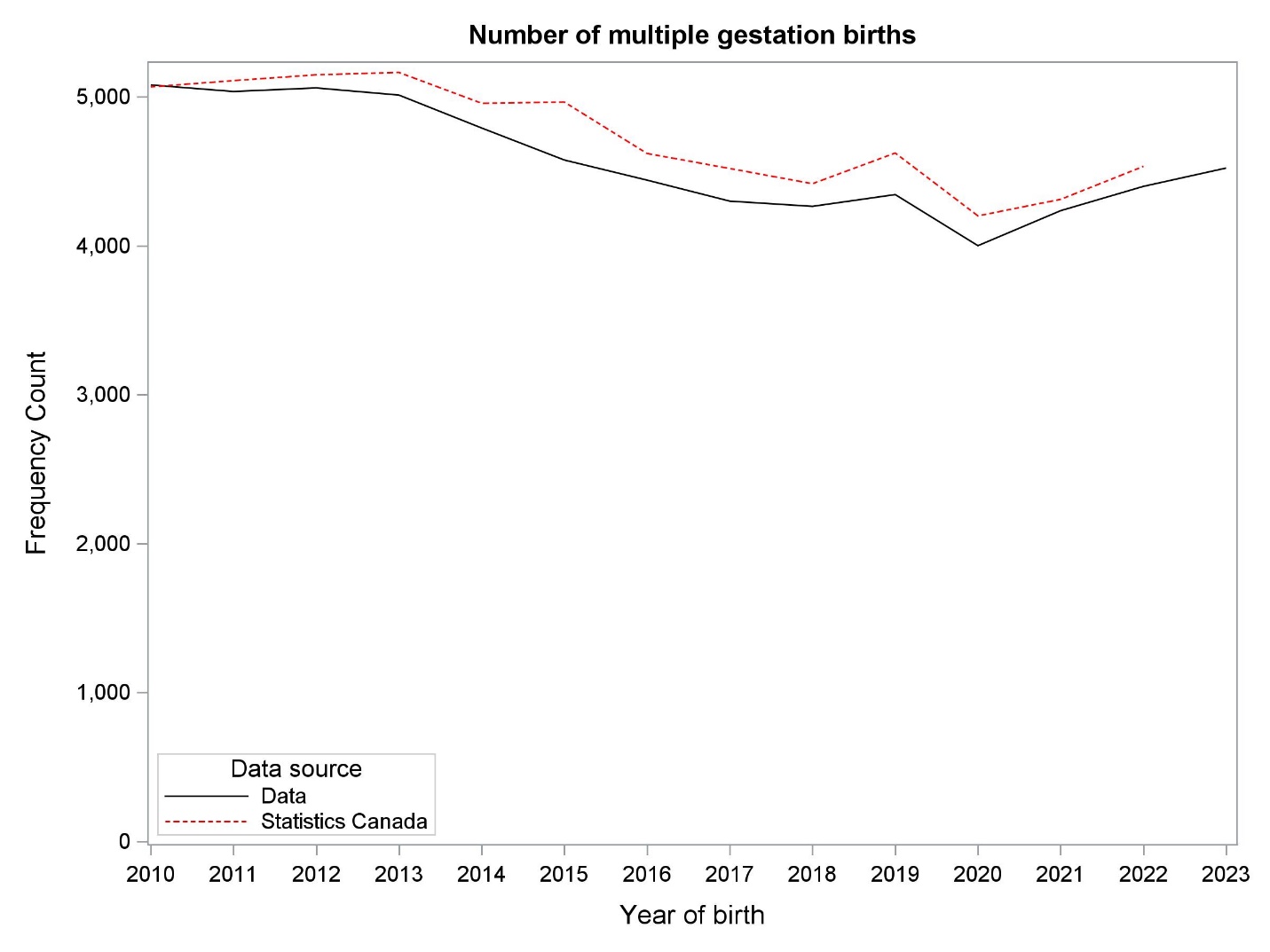


The number of stillbirths was more substantially underreported, with approximately 50% of cases reported by Statistics Canada being captured by the inpatient database. It is possible that several stillborns were instead classified as liveborns or underreported in hospital records if considered non-viable (Fell et al., 2020). Further work is needed to understand the underlying reasons for the under-capture of stillborns.


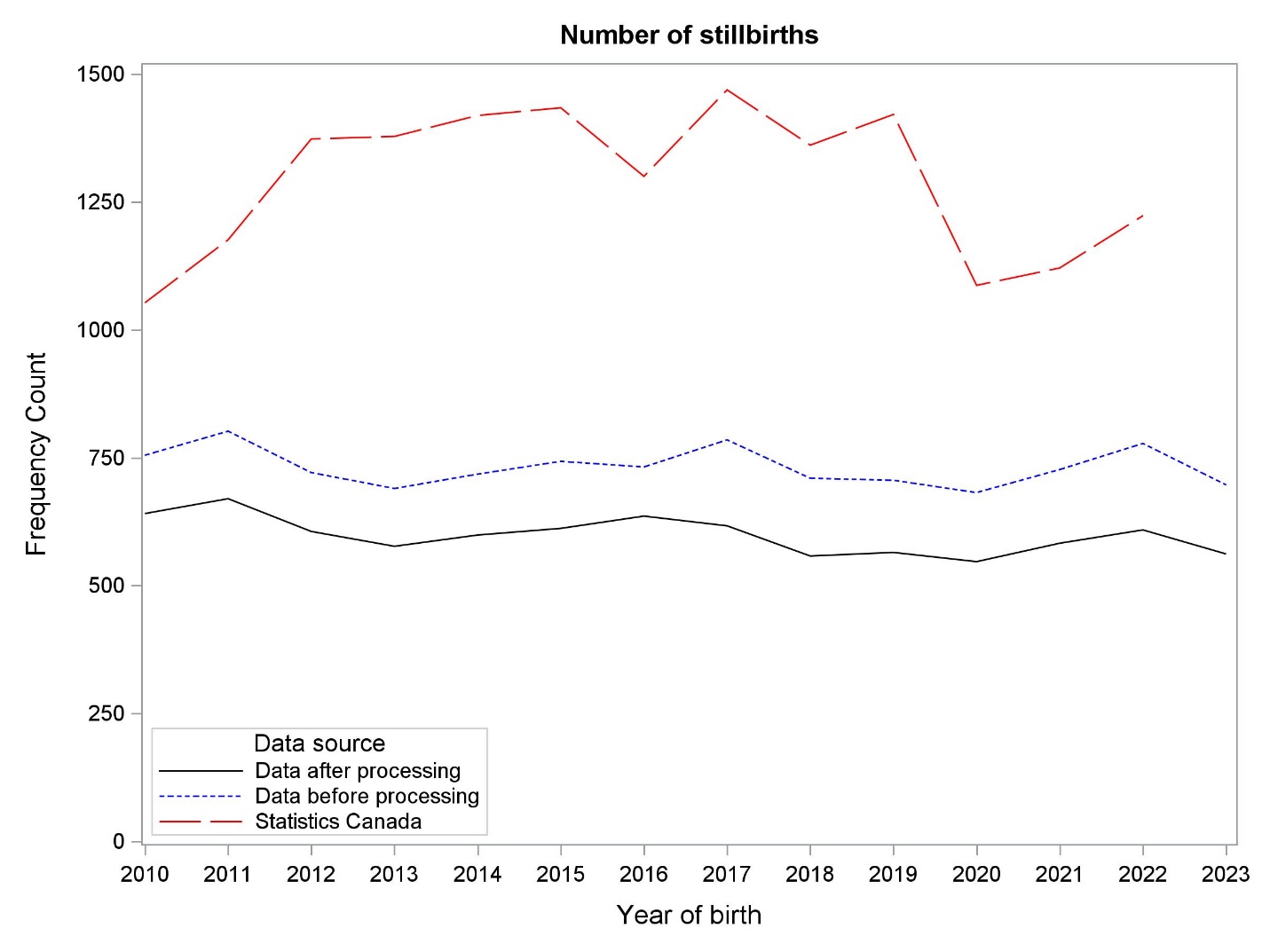


# References

1. Male V. SARS-CoV-2 infection and COVID-19 vaccination in pregnancy. Nat Rev Immunol. 2022 May 1;22(5):277–82.

2. Wei SQ, Bilodeau-Bertrand M, Liu S, Auger N. The impact of COVID-19 on pregnancy outcomes: a systematic review and meta-analysis. CMAJ. 2021 Apr 19;193(16):E540–8.

3. Regan AK, Arah OA, Fell DB, Sullivan SG. SARS-CoV-2 Infection During Pregnancy and Associated Perinatal Health Outcomes: A National US Cohort Study. J Infect Dis. 2022 Mar 1;225(5):759–67.

4. Villar J, Ariff S, Gunier RB, Thiruvengadam R, Rauch S, Kholin A, et al. Maternal and Neonatal Morbidity and Mortality Among Pregnant Women With and Without COVID-19 Infection: The INTERCOVID Multinational Cohort Study. JAMA Pediatr. 2021 Aug 1;175(8):1.

5. Khoury JE, Atkinson L, Bennett T, Jack SM, Gonzalez A. Prenatal distress, access to services, and birth outcomes during the COVID-19 pandemic: Findings from a longitudinal study. Early Hum Dev. 2022 Jul 1;170.

6. Roberts NF, Sprague AE, Taljaard M, Fell DB, Ray JG, Tunde-Byass M, et al. Maternal-Newborn Health System Changes and Outcomes in Ontario, Canada, During Wave 1 of the COVID-19 Pandemic-A Retrospective Study. J Obstet Gynaecol Can. 2022 Jun 1;44(6):664–74.

7. Hospital Harm Indicator General Methodology Notes [Internet]. Ottawa, ON: Canadian Institute for Health Information; 2021 [cited 2023 May 23]. Available from: https://www.cihi.ca/sites/default/files/document/hospital-harm-indicator-general-methodology-notes.pdf

8. Kramer MS, Platt RW, Wen SW, Joseph KS, Allen A, Abrahamowicz M, et al. A new and improved population-based Canadian reference for birth weight for gestational age. Pediatrics. 2001;108(2).

9. Home | Agency for Healthcare Research and Quality [Internet]. [cited 2022 Sep 28]. Available from: https://www.ahrq.gov/

10. Hospitalization and Childbirth Quick Stats Metadata [Internet]. Ottawa, ON: Canadian Institute for Health Information; 2022.

11. Joseph KS, Fahey J, Canadian Perinatal Surveillance System. Validation of perinatal data in the Discharge Abstract Database of the Canadian Institute for Health Information. Chronic Dis Can. 2009;29(3):96–100.

12. A Guide to Obstetrical Coding [Internet]. Ottawa, ON: Canadian Institute for Health Information; 2022.

13. Institute of Medicine (US) Committee on Understanding Premature Birth and Assuring Healthy Outcomes. Measurement of Fetal and Infant Maturity. In: Behrman RE, Butler AS, editors. Preterm Birth: Causes, Consequences, and Prevention. Washington (DC): National Academies Press (US); 2007.

14. Matheson FI, Dunn JR, Smith KLW, Moineddin R, Glazier RH. Development of the Canadian Marginalization Index: a new tool for the study of inequality. Can J Public Health. 2012;103(8 Suppl 2).

15. Snelgrove JW, Simpson AN, Sutradhar R, Everett K, Liu N, Baxter NN. Preeclampsia and Severe Maternal Morbidity During the COVID-19 Pandemic: A Population-Based Cohort Study in Ontario, Canada. J Obstet Gynaecol Can. 2022 Jul 1;44(7):777–84.

16. Molina RL, Tsai TC, Dai D, Soto M, Rosenthal N, Orav EJ, et al. Comparison of Pregnancy and Birth Outcomes Before vs During the COVID-19 Pandemic. JAMA Netw open. 2022 Aug 12;5(8):E2226531.

17. Gu J, Karmakar-Hore S, Hogan ME, Azzam HM, Barrett JFR, Brown A, et al. Examining Cesarean Section Rates in Canada Using the Modified Robson Classification. J Obstet Gynaecol Can. 2020 Jun 1;42(6):757–65.

18. Zhao Y, Zhang J, Hukkelhoven C, Offerhaus P, Zwart J, De Jonge A, et al. Modest Rise in Caesarean Section from 2000-2010: The Dutch Experience. PLoS One. 2016 May 1;11(5).

19. Discharge Abstract Database Open-Year Data Quality Test Specifications, 2014–2015 [Internet]. Ottawa, ON: Canadian Institute for Health Information; 2015.

20. Gurol-Urganci I, Waite L, Webster K, Jardine J, Carroll F, Dunn G, et al. Obstetric interventions and pregnancy outcomes during the COVID-19 pandemic in England: A nationwide cohort study. PLoS Med. 2022 Jan 1;19(1).

21. Melov SJ, Elhindi J, Mcgee TM, Lee VW, Cheung NW, Chua SC, et al. Investigating service delivery and perinatal outcomes during the low prevalence first year of COVID-19 in a multiethnic Australian population: a cohort study. BMJ Open. 2022 Jul 1;12(7).

22. Montoya-Williams D, Lemas DJ, Spiryda L, Patel K, Neu J, Carson TL. What Are Optimal Cesarean Section Rates in the U.S. and How Do We Get There? A Review of Evidence-Based Recommendations and Interventions. J Women’s Heal. 2017 Dec 12;26(12):1285.

23. Neu J, Rushing J. Cesarean versus vaginal delivery: long-term infant outcomes and the hygiene hypothesis. Clin Perinatol. 2011 Jun;38(2):321–31.

24. Bohren MA, Opiyo N, Kingdon C, Downe S, Betrán AP. Optimising the use of caesarean section: a generic formative research protocol for implementation preparation. Reprod Health. 2019 Nov 19;16(1).

25. Betrán AP, Ye J, Moller AB, Zhang J, Gülmezoglu AM, Torloni MR. The Increasing Trend in Caesarean Section Rates: Global, Regional and National Estimates: 1990-2014. PLoS One. 2016 Feb 1;11(2).

26. Crowley CM, Lang NA, O’Leary BD, Geary MP. Trends in instrument preference for operative vaginal delivery in a tertiary referral center: 2008-2021. Int J Gynaecol Obstet. 2023 Mar 10;

27. Merriam AA, Ananth C V., Wright JD, Siddiq Z, D’Alton ME, Friedman AM. Trends in operative vaginal delivery, 2005-2013: a population-based study. BJOG. 2017 Aug 1;124(9):1365–72.

28. Dutywa A, Olorunfemi G, Mbodi L. Trends and Determinants of Operative Vaginal Delivery at Two Academic Hospitals in Johannesburg, South Africa 2005-2019. Int J Environ Res Public Health. 2022 Dec 1;19(23).

29. Di Toro F, Gjoka M, Di Lorenzo G, De Santo D, De Seta F, Maso G, et al. Impact of COVID-19 on maternal and neonatal outcomes: a systematic review and meta-analysis. Clin Microbiol Infect. 2021 Jan 1;27(1):36–46.

30. Live births and fetal deaths (stillbirths), by place of birth (hospital or non-hospital) [Internet]. [cited 2023 May 9]. Available from: https://www150.statcan.gc.ca/t1/tbl1/en/tv.action?pid=1310042901

31. Dvash S, Cuckle H, Smorgick N, Vaknin Z, Padoa A, Maymon R. Increase rate of ruptured tubal ectopic pregnancy during the COVID-19 pandemic. Eur J Obstet Gynecol Reprod Biol. 2021 Apr 1;259:95.

32. Peles G, Paz-Levy D, Wainstock T, Goldbart A, Kluwgant D, Sheiner E. Pediatric respiratory hospitalizations in small for gestational age neonates born at term. Pediatr Pulmonol. 2022 Mar 1;57(3):754–60.

33. Martín-Calvo N, Goni L, Tur JA, Martínez JA. Low birth weight and small for gestational age are associated with complications of childhood and adolescence obesity: Systematic review and meta-analysis. Obes Rev. 2022 Jan 1;23 Suppl 1(S1).

34. Zietlow AL, Nonnenmacher N, Reck C, Ditzen B, Müller M. Emotional Stress During Pregnancy - Associations With Maternal Anxiety Disorders, Infant Cortisol Reactivity, and Mother-Child Interaction at Pre-school Age. Front Psychol. 2019 Sep 1;10(SEP).

35. Kyrgios I, Giza S, Tsinopoulou VR, Maggana I, Haidich AB, Galli-Tsinopoulou A. Seasonality of month of birth in children and adolescents with autoimmune thyroiditis: a continuing conundrum. J Pediatr Endocrinol Metab. 2018 Oct 1;31(10):1123–31.

36. González-Leonardo M, Rowe F, Fresolone-Caparrós A. Rural revival? The rise in internal migration to rural areas during the COVID-19 pandemic. Who moved and Where? J Rural Stud. 2022 Dec 1;96:332–42.
