# Supplementary material for: Temporal-spatial trends in childbirth in Ontario, Canada
Source: PLoS One. 2026 Mar 30;21(3):e0342215. doi: 10.1371/journal.pone.0342215 (PMC13035117; doi:10.1371/journal.pone.0342215)
Supplement: Attachment — Submitted filename: Response to reviewers - labor delivery.docx [file pone.0342215.s005.docx]

March 17, 2025

**Re: PONE-D-24-52959** “Temporal-spatial trends in labor and delivery in Ontario”

Dear Dr Desseauve,

On behalf of all authors, we thank you and the reviewers for the review and suggestions to improve the manuscript. We provide point-by-point responses below. Any other changes were to meet PLOS ONE’s stylistic requirements. We believe the manuscript has been strengthened following these revisions.

Thank you for your time and consideration

Sincerely,

Steven Habbous, PhD

Epidemiologist

Email: steven.habbous@ontariohealth.ca

**Reviewer Comments:**


**Reviewer #1:**

Review: temporal spatial trends in labor and delivery in Ontario
Thank you for having me review this work that describe temporal-spatial trend in socio-demographics, clinical factors, care patterns and perinatal outcomes over years. Here are some remarks:

*Introduction*

**Comment 1:** The overall aim of the study is clear. However, the introduction is very succinct (only 2 references) and, in my opinion, lacks contextual elements.

**Response:** Thank you for the suggestion. We provide additional context in the introduction as it relates to the relevance of the broad approach of the study. We also extended the introduction to align with comments 2 and 3 below.

**Comment 2:** It would be interesting to address the impact of changes in women's socio-demographic and clinical characteristics (e.g. age, obesity, diabetes, hypertensive disorders) on practices (such as caesarean section) and maternal and neonatal morbidity and mortality.

**Response:** We added the sentence below to the introduction to frame the importance of the “broad-ranging longitudinal perspective” of the study.

“This is particularly useful because understanding how maternal characteristics have changed over time (e.g., age at delivery, presence of comorbidities) may influence medical decision-making and perinatal outcomes (PMID 39366197). The literature from an Ontario setting appears to be sparse, but Canadian evidence suggests increasing rates of comorbidities over time including preeclampsia, gestational diabetes, glucose disorders, overweight/obesity (37948551, 21184681).”

**Comment 3:** You also mentioned assessing the impact of the COVID 19 pandemic in the objective of the study. It would be interesting to give more details on how this pandemic may have affected perinatal health outcomes.

**Response:** We include additional references around the potential impact of the COVID-19 pandemic on 1) healthcare utilization generally; and 2) perinatal outcomes associated with SARS-CoV-2 infection. It reads:

“Previous work has shown delayed and reduced prenatal care, fewer post-partum visits, and higher rates of comorbidity during the early pandemic (PMID 40075540). Most studies tended to compare perinatal outcomes among pregnant persons who also tested positive for SARS-CoV-2 infection, demonstrating worse outcomes among those testing positive (PMID 35282784) or had short follow-up limited to the first year of the pandemic (PMID 34344771).

**Comment 4:** Could you also specify whether other work of this type has already been carried out in Canada, and give an estimate of maternal and neonatal mortality? Are any of these indicators collected at national level?

**Response:** We have further reviewed the literature and added references above from a Canadian setting. Several indicators are indeed collected and reported at a national level (https://www.cihi.ca/en/access-data-and-reports/data-tables) and we used these statistics to help validate the linkages we used to measure these indicators. Two key data tables are:

1. Canadian Institute for Health Information. *Hospitalization and Childbirth, 1995–1996 to 2023–2024 — Supplementary Statistics*. Ottawa, ON: CIHI; 2025.
2. Canadian Institute for Health Information. *Inpatient Hospitalization, Surgery and Newborn Statistics, 2023–2024*. Ottawa, ON: CIHI; 2025.

**Comment 5:** Were there any major changes (excluding the COVID 19 pandemic) in perinatal health policies or care services during the study period? If so, please give some contextual information.

**Response:** This is an interesting question. A review of the literature revealed a few local or small interventions, but not much beyond this. We have added these to the introduction:

“Apart from the COVID-19 pandemic, we are unaware of any provincial changes in protocols or standards that would have largely influenced perinatal outcomes, with the potential exception of the creation of two stand‐alone midwifery‐led birth centers in Toronto and Ottawa in 2014 [9].”

*Methods*

**Comment 6:** What is the proportion of hospital births in Ontario?

**Response:** From our administrative data assets, we cannot reliably estimate the total number of births in Ontario. The out-of-hospital flags do not represent all out-of-hospital births. Using the grey literature, the percentage of home births in Ontario is estimated to be ~15%, decreasing from 19% in the 2010/11 fiscal year to 13% in the 2019/20 fiscal year (https://www.ontariomidwives.ca/midwiferydatamatters-examining-rise-out-hospital-birth-during-covid-19-pandemic.) We added this information to the “Additional Inclusions and exclusions” subsection of the Methods:

**“**Out-of-hospital births (whether planned or unplanned) were omitted because the out-of-hospital flags do not represent all out-of-hospital births. The percentage of out-of-hospital births in Ontario is estimated to be 10-20% (reference).”

**Comment 7:** To clarify the study population from the outset, you may wish to state clearly at the start of the method that you are considering only births to women residing in Ontario during the study period, live births and hospital births. The methodological details come later, and concern the strategy for selecting this population.

**Response:** Thank you for the suggestion. The first sentence of the “Cohort Creation” subsection of the methods now reads:

“The present study includes only Ontario residents who delivered liveborn in an Ontario hospital between 2010 and 2023.”

**Comment 8:** Please detail the definition of all ICD codes, or consider giving them as appendix

**Response:** We have added these to supplementary tables S2 (procedure codes) and S3 (ICD10 codes).

**Comment 9:** It's not clear how the different ICD10 codes were selected. Is it to identify the study population? Is “-“(O10-O16) signify “from O10 to O16”, all selected ? What is the signification of “with a 6th digit of 1 or 2?

**Response:** In the present revision, in the cohort creation subsection of the methods, we now state “The cohort was created using ICD10 codes based on the methodology provided by CIHI [3].” We now also clarify that the ranges are inclusive (your interpretation is correct), and indicate the significance of the 6^th^ digit of 1 (delivered) and 2 (delivered with complications).

**Comment 10:** You might specify first the identification strategy and then the method adopted (for example, to identify delivery-associated abstract, we looked for women with a diagnosis of childbirth or any other pregnancy-related diagnosis. For this, we searched for the following ICD-10 codes…etc). To identify birth-associated abstract with a live newborn, we looked for …etc.). Please consider using the same terms for clarity (newborn abstract / birth associated abstract / birth-associated records – delivery discharge abstract / delivery associated abstract / delivery associated recode).

**Response:** Thank you for the suggestions. We agree that this approach makes it easier to follow. We adapted your wording directly in this part of the methods section. We have gone through the write-up as well to ensure consistency in the use of terminology (we chose to use the terms delivery abstracts and newborn abstracts).

**Comment 11:** The first three sentences of the “matching” paragraph do not correspond to the matching strategy. You could move them to the previous paragraph in connection with the abstract identification and selection strategy.

**Response:** We agree and have moved those three paragraphs to the previous subsection of the methods.

**Comment 12:** the “additional inclusions and exclusions” paragraph could be called exclusions only (I don't see any notion of additional inclusions)?

**Response:** Thank you for noticing this, we have renamed this subheading to “Exclusions”

**Comment 13:** I don't understand the sentence (the number of live-born deliveries was comparable, but only before the application of additional exclusions).

**Response:** I agree this is awkwardly phrased. We removed the part in the parentheses.

**Comment 14:** Regional variability: this part should be on the “statistics” part.

**Response:** We agree and have move this to the statistics subsection of the methods.

**Comment 15:** Please detail the definition of birth or obstetrical trauma.

**Response:** We now have the detailed descriptions of all the ICD10 codes used in the supplement, so these details are now available. In the methods, we describe obstetrical trauma as including lacerations or uterine rupture. We now also describe birth trauma as injuries to the newborn.

**Comment 16:** For indirect standardisation to compute the rate of delivery over time, why did you choose the year 2019 as reference? The denominators were all population or only women? To explain this method, you could point out that it allows to take into account any differences in the age structure of the Ontario population over time. To be more precise, please indicate that you compute the age-standardised in-hospital births incidence ratio (not the rate of delivery over time).

**Response:** Thank you for your question. The choice of 2019 as the reference year was made because it was the most recent year before the pandemic. In the revision, we clarify that the denominators were only women. We have incorporated your suggested wording as well, as it is more accurate.

**Comment #17:** Why is multiparity or unknown parity considered as a complex characteristic of pregnancies? I was surprised by the percentage of pregnancies considered complex (around 70%).

**Response:** Thank you for raising this point. The Canadian Institute for health Information defines “low-risk pregnancies” which formed the basis for the definition of “complex”. We acknowledge that this definition of complexity has likely changed over time and may vary from one hospital to the next. The multiparity or unknown parity is “complex” relatively to “low-risk pregnancies” by this definition. The multiparity or unknown parity might not be complex pregnancies under other conditions or context. We have explained it and added the following to the limitations section of the discussion:

**“**Another limitation is the definition of a complex pregnancy. We acknowledge that this is sometimes subjective and may change over time and vary by hospital. Broadly speaking, a complex pregnancy is any pregnancy where there is an elevated risk of adverse outcomes to the newborn or PWD, but what constitute “elevated risk” and “adverse outcomes” are not well defined (PMID 34175656). The multiparity or unknown parity is “complex” relatively to “low-risk pregnancies” by this definition. The multiparity or unknown parity might not be complex pregnancies under other conditions or context. Classifying a pregnancy as complex should have implications on medical decision-making and provision of care (e.g., use of Cesarean delivery; frequency of neonatal blood glucose monitoring).”

**Comment 18:** You fixed your statistical significance set at 1%. Why this choice (I understand that due to the large sample size you had to lowered it but why 1% and not lower?) How was this choice made?

**Response:** Considering the large sample size, we wanted to keep our type 1 error rate to be minimum. The choice of p-value threshold was arbitrary, but 1% is the next most commonly chosen threshold (from experience) less than 5%.

*Results*

**Comment 19:** Table 1 is very heavy. Couldn't you consider presenting, for example, the most prevalent complexity characteristics in table 1 and the others in an appendix? You could also split it into 2 tables. For continuous variable, consider describe them either as mean(sd) or median(IQR) depending on the distribution (but not both).

**Response:** Thank you for the suggestion. We have split the table into four and remove either mean (SD) or median (IQR) where appropriate:
Table 1: Sociodemographic characteristics over time
Table 2: Clinical characteristics over time
Table 3: Healthcare utilization patterns over time
Table 4: Clinical outcomes over time

**Comment 20:** Could you describe the rate of obstetric haemorrhage overall (not only for assisted and non-assisted vaginal delivery respectively) ?

**Response:** We have added these statistics. For completeness, we also added this for obstetrical hemorrhage.

**Comment 21:** Fig 2: The label for the Y axis (age-standardized in-hospital births incidence ratio) must be specified.

**Response:** We have made this change

**Comment 22:** Some results are redundant between Table 1 and Figures. It is not useful to present % over time in both figure and table for the same variables. More generally, there are a lot of results and figures. Consider synthesizing to make your message clearer. For example, I wonder whether the impact of the covid 19 pandemic should be addressed in another work.

**Response:** To strike a balance, we have moved Table 2 (Clinical characteristics over time), Table 3 (Healthcare utilization patterns over time), and Table 4 (Clinical outcomes over time) to the supplement. We find visualizations are a more efficient means to convey information, but interested readers can refer to the appendix for the actual estimates. We have opted to retain the results related to the COVID-19 pandemic because this was an impactful aspect affecting temporal changes in healthcare utilization patterns (and possibly outcomes).

**Comment 23:** I don't understand the relevance of presenting the covid period in the graphs for maternal characteristics.

**Response:** Agreed, we have removed this aspect of Figure 3

**Comment 24:** The first sentence of the regional variability in outcomes paragraph should be presented in the methods section. The healthcare utilization variables analysed should also be detailed in the methods section. You called them regional variability but to me its more hospital level variability as the denominator is the number of deliveries for each hospital.

**Response:** We have moved this sentence to the methods section (combined with comment #14 above) and have renamed it as “hospital level variability” instead of “regional variability” throughout the manuscript.

**Comment #25:** The use of funnel plots and their reading should be further developed. They are not easy to interpret.

**Response:** In the methods section, we have added interpretative advice for the funnel plots and references as well:

“Restricted to the most recent time period (2020-2023), we examined hospital-level variability in select measures of healthcare utilization (C-section, midwife involvement, epidural administration) using funnel plots. Funnel plots provide a visual glimpse of proportions while taking into consideration the size of the denominators [28]. We provide a 95% and 99% confidence band for the proportions. Hospitals above (below) the confidence band have a higher (lower) proportion than the population mean for a hospital with its specific volume of deliveries.”

**Comment 26:** Why isn't the denominator for funnel plots 5 the total number of deliveries (as for other funnel plots, number of delivery (2020-2023))? If not, the text should specify that it's not just about smaller / higher volume hospitals, but also about those with fewer / higher complex (5A) or non-situations (5B).

**Response:** Thank you for raising this. The x-axis for the funnel plot corresponds to the denominator of the indicator, so they are different for each funnel plot. Your interpretation is correct, and we have re-worded the results section to read as follows:

“Hospitals that performed a smaller number of complex deliveries were less likely to deliver via C-section than hospitals performing a greater number of complex deliveries, but no such pattern emerged for non-complex deliveries (**Fig 5A-B**). Pregnant individuals delivering at smaller-volume hospitals were more likely to involve a midwife (**Fig 5C**). Hospitals performing fewer vaginal deliveries were less likely to administer an epidural than hospitals performing more vaginal deliveries (**Fig 5D**).”

**Comment #27:** I'm not sure that figure S2 and these results stratified on SarsCoV2 status are of much interest.

**Response:** We agree and have removed this supplementary figure and its reference from the paper.

*Discussion*

**Comment #28:** LOS for length of stay has not been defined previously

**Response:** Thank you for noticing this, we have defined LOS at its first mention.

**Comment #29:** I'm not convinced by your paragraph discussing the rising C-section rate. If this were essentially explained by the rise in complex pregnancies, you would observe, by stratifying the analyses, an overall increase in caesarean section rates for complex pregnancies, but no increase over time. Are you suggesting that among complex pregnancies, the level of complexity has increased over time? If not, what other factors might explain this increase in C-section rates? Are any strategies being considered to reduce them?

**Response:** Thank you for raising this point. The rise in C-section rate was small, and predominantly observed at the onset of the COVID-19 pandemic. We are unaware of any strategies to reduce C-section delivery rate, and given the focus away from using this as a quality indicator, there is unlikely to be a strategy. We restated this paragraph in the discussion to read as follows:

“The optimal rate of C-section delivery has previously been suggested to be between 15% and 19%, a rate exceeded by most high-income nations [29,30]. Internationally, the increasing use of C-section delivery was accelerated during the pandemic [31–37]. We also found a small increase in the use of C-section delivery following the pandemic, regardless of complexity categorization. Reasons for these trends are unclear, but C-section delivery rate is a suboptimal process indicator of healthcare quality [38].”

**Comment #30:** You don't mention the reduction in infant mortality over time. Has this been demonstrated previously, and what factors might explain this positive result?

**Response:** great point, we have added the following paragraph to the discussion and a more useful figure to make the trends more obvious (now 6B, pasted below):

“Although rare, mortality after delivery increased over the study period while mortality of the newborn decreased. The majority of deaths occurring within 1 year of birth occurred within the first 6 months. Reasons for decreasing newborn mortality may be driven by improvements in prenatal care, uptake of prenatal screening, and increasing access to medical abortion that may be differentially used to terminate fetuses having a higher risk of mortality or improvement in neonatal and infant care [52,53]. In contrast to newborns, deaths occurring within 1 year of delivery were more evenly distributed over time (e.g., approximately half occurred in the first 6 months). Thus, reasons for post-delivery mortality may extend beyond birthing-related complications, including preexisting conditions and post-partum mental health disorders [55–57].”

**Comment #31:** Several elements presented in the strengths part of the manuscript are elements of discussion (rate of epidural and travel distance).*

**Response:** Thank you for noting this, we have removed the subheading “strength” from the discussion.

**Comment #32:** Could you looked at emergency vs planned caesarean with your data?

**Response:** This is an interesting question. It seems that there is no validated approach to distinguish emergency from planned caesarean using our data. One study used a creative approach to achieve this, selecting healthy women with a singleton breech presentation having evidence of labor (https://www.cmaj.ca/content/176/4/455). This should be an area for further research and validation.
