# Supplementary material for: Temporal-spatial trends in childbirth in Ontario, Canada
Source: PLoS One. 2026 Mar 30;21(3):e0342215. doi: 10.1371/journal.pone.0342215 (PMC13035117; doi:10.1371/journal.pone.0342215)
Supplement: Attachment — Submitted filename: Response to reviewers - labor delivery - PONE-D-24-52959.R2.docx [file pone.0342215.s006.docx]

June 13, 2025

**Re: PONE-D-24-52959.R1** “Temporal-spatial trends in labor and delivery in Ontario”

Dear Dr Desseauve,

Thank you for the additional suggestions to bolster the discussion. On behalf of all authors, we thank you for your consideration.

Sincerely,

Steven Habbous, PhD

Epidemiologist

Email: steven.habbous@ontariohealth.ca

**Additional Editor Comments:**

I find that the authors have addressed the majority of the reviewer comments with care and diligence. The introduction has been meaningfully expanded to provide a stronger contextual foundation, and the methods section now includes sufficient technical detail and transparency regarding cohort creation, data linkage, and indicator definitions. The restructuring of tables and streamlining of figures improve clarity, and the decision to relocate more granular data to the supplement is appropriate.

Moreover, the discussion appropriately reflects on the policy and clinical relevance of the findings while acknowledging the inherent limitations of administrative data.

**Comment:** While a few minor clarifications could further strengthen the manuscript (e.g., deeper discussion of alternative explanations for the rise in cesarean delivery or stratified outcomes by complexity subgroups)

**Response:** Thank you for the suggestion. We have added additional thoughts and references to the discussion to potentially explain the observed rise in cesarean delivery over time.

The optimal rate of C-section delivery has previously been suggested to be between 15% and 19%, a rate exceeded by most high-income nations [27,28]. Internationally, the increasing use of C-section delivery was accelerated during the pandemic [29–35]. We also found a small increase in the use of C-section delivery following the pandemic, regardless of complexity categorization. Reasons for these trends are unclear, but may be driven by a rise in unscheduled cesarean delivery associated with increasing maternal age (PMID 37062508); increasing incidence of maternal or fetal complications such as fetal distress, slow progress in labor, breech presentation, and repeat cesarean section (PMC4126949, 34971814, 31976544, 34971814); or increasing acceptance among patients and providers that C-section delivery is a normal mode of delivery (PMC9049329, 37216058). Hospital culture has also been demonstrated to impact the rate of primary cesarean delivery (PMC6407356). Our observation that lower-volume hospitals tended to provide epidurals less frequently or perform fewer C-section deliveries for complex pregnancies supports a regional effect that may be driven by hospital culture, patient preferences, or resource availability. Although the C-section delivery rate is a suboptimal process indicator of healthcare quality on its own, more details are needed on their indications to enable appropriate contextualization [36].
