# Supplementary material for: Temporal-spatial trends in childbirth in Ontario, Canada
Source: PLoS One. 2026 Mar 30;21(3):e0342215. doi: 10.1371/journal.pone.0342215 (PMC13035117; doi:10.1371/journal.pone.0342215)
Supplement: Attachment — Submitted filename: Response to reviewers - labor delivery - PONE-D-24-52959.R3.docx [file pone.0342215.s007.docx]

February 13, 2026

**Re: PONE-D-24-52959.R2** “Temporal-spatial trends in labor and delivery in Ontario”

Dear Dr Campbell,

On behalf of all authors, we thank you and the reviewers for the review and the additional suggestions to improve the manuscript. On behalf of all authors, we thank you for your consideration.

Sincerely,

Steven Habbous, PhD

Epidemiologist

Email: [shabbous@uwo.ca](mailto:shabbous@uwo.ca); steven.habbous@ontariohealth.ca

**Reviewer Comments**

**Reviewer #1:**

**Comment #1:** The authors have fully addressed the reviewers' and editor's requests to improve their manuscript for publication.

**Response:** Thank you.

**Reviewer #2:**

Reviewer #2: Many thanks for the opportunity to review the manuscript “Temporal-spatial trends in labor and delivery in Ontario,” which addresses an important topic in the current political climate. However, the study may be of interest to a relatively limited group of stakeholders.

**Comment #1:** The title does not appear to align with the manuscript’s content. I could not identify any data related to “labor,” as the analysis focuses on “delivery.” Using the term “childbirth” may provide a more accurate description.

**Response:** Thank you for the suggestion, we have retitled the manuscript to read “Temporal-spatial trends in childbirth in Ontario”.

**Comment #2:** From the abstract it reads: Conclusion: Over the last 14 years, we found an increasing incidence of people giving birth having more complex demographic and clinical characteristics. How do the authors define “complex demographic”? And which data are this statement based on?

**Response:** Thank you for noticing this, we agree that this is unclear. The part about demographics relates to age, but the part about clinical characteristics refers to conditions like gestational diabetes, obesity, pre-eclampsia, etc. We rephrased the conclusion to read: “Over the last 14 years, we found an increasing incidence of people giving birth at an older age and having complicating clinical characteristics at the time of delivery.”

**Comment #3:** It reads in the manuscript “Understanding the continuum of care throughout the perinatal period is important for health system monitoring and quality improvement”. Perinatal period standard definition is from 22 completed weeks of gestation (154 days) to 7 completed days after birth but the authors go beyond this period. Why or do the authors define the perinatal period differently?

**Response:** Thank you for pointing this out. As we delve further into this space, it would be important to clearly articulate and define the time-periods used, although there is some heterogeneity in the literature particularly around the post-partum period (PMC11051636). In this revision, we introduce the term postpartum and try to avoid using the terms perinatal and postpartum interchangeably. In the methods, we describe measures of healthcare utilization and delivery and outcomes specific to the perinatal period.

**Comment #4:** Why did the study only include liveborn deliveries (vaginal or caesarean) and not considering stillbirths? It would be very important to include stillbirth as well.

**Response:** We agree that this is a limitation and is a current methodological issue that we are keen to resolve. The limitations section of the discussion reads as follows:

“Another limitation is a lack of information on stillbirths. PWD in-hospital with a stillborn delivery may be at higher risk than those delivering liveborn, but we were only capturing half of the expected number of stillbirths in the province (**Technical Appendix**). For population-based health system monitoring, PWD stillborn should be considered.”

**Comment #5:** Please add a reference to this statement: In this study, we take a broad-ranging and longitudinal perspective to examine long-term changes in the quality of obstetric care in Ontario, representing 40% of all births in Canada.

**Response:** We have added the following reference:

Statistics Canada. [Table 13-10-0414-01  Live births, by place of residence of mother](https://www150.statcan.gc.ca/t1/tbl1/en/tv.action?pid=1310041401)
**DOI:** <https://doi.org/10.25318/1310041401-eng>

**Comment #6:** Why using the cut-off of 6 months mortality (from the manuscript: We measured maternal and neonatal mortality within 6 months of delivery/birth) as the authors are interested in the perinatal period according to the manuscript? The authors report on unplanned emergency department (ED) visits within 42 days of delivery or birth. Why 42 days if the focus of the analysis is during the perinatal period? I find it a bit confusing using “….visits within 42 days of delivery or birth” instead of saying postpartum.

**Response:** In the present revision we have made the distinction between the perinatal period and the postpartum period. The literature also seems to be inconsistent on whether to report mortality and ED visits at 42 days, 6 months, or 1 year (we have found all three), so we follow suit to be consistent with this.

**Comment #7:** Not sure I understand this paragraph “Funnel plots provide a visual glimpse of proportions while taking into consideration the size of the denominators [26]. We provide a 95% and 99% confidence band for the proportions. Hospitals above (below) the confidence band have a higher (lower) proportion than the population mean for a hospital with its specific volume of deliveries”.

**Response:** We have simplified this description to read as follows:

“Funnel plots provide a visual glimpse of proportions while taking into consideration the size of the denominators [26]. We provide a 95% and 99% confidence band for the proportions. Hospitals above (below) the confidence band have a higher (lower) proportion than the mean”.

**Comment #8:** Please add labels to Table 1 Table: Trends of sociodemographic characteristics over time. Not clear for the reader to understand how Material resources are defined as well as Racialized/newcomer.

**Response:** Thank you, we have indicated that these are quintiles

**Comment #9:** Fig 2: Age-standardized incidence ratio of the number of births. Rates were indirectly standardized to the rate in 2019. Is this ratio or rate?

**Response:** Thank you for your question. We plotted age-standardized incidence ratios (although the rates are implicitly used to compute them). To make the estimates more useful to other readers who wish to compare their own rates to the same reference population, we switched to direct standardization using the 2025 female population. The new figure looks similar to the previous one, except the y-axis is now a rate instead of a ratio and the legend is updated:

**Fig 2: Age-standardized incidence rate of the number of births performed in-hospital.** Using direct standardization, age-specific rates were applied to the female population in Canada from 2025.

**Comment #10:** Why did the authors not apply the Robson Classification (<https://robson-classification-platform.srhr.org/about>)?

**Response:** Thank you for the question. We did not want to comment on the appropriateness of C-section delivery and the Robson score was not available in our data until 2017. This should be the focus of a future study, and we await some more detailed information as our data assets mature (see next comment).

**Comment #11:** The authors write: Access to comprehensive data are needed to encourage quality improvement investigations. Suppose not only a matter of access, are the data collected?

**Response:** We believe much of the data are collected by BORN (Better Outcomes Registry & Network). However, we do not (yet) have access to these data and are hoping to be able to access it for future linkage and more in-depth analysis.

**Comment #12:** Please provide an abbreviation list.

**Response:** We have added this and reduced the use of abbreviations throughout.

Thank you for all your suggestions and for taking the time to review!
